# Test-Retest Reliability of “High-Order” Functional Connectivity in Young Healthy Adults

**DOI:** 10.3389/fnins.2017.00439

**Published:** 2017-08-02

**Authors:** Han Zhang, Xiaobo Chen, Yu Zhang, Dinggang Shen

**Affiliations:** ^1^Department of Radiology and Brain Research Imaging Center, University of North Carolina at Chapel Hill Chapel Hill, NC, United States; ^2^Department of Brain and Cognitive Engineering, Korea University Seoul, South Korea

**Keywords:** test-retest, reliability, functional connectivity, high-order connectivity, resting-state fMRI, dynamic connectivity

## Abstract

Functional connectivity (FC) has become a leading method for resting-state functional magnetic resonance imaging (rs-fMRI) analysis. However, the majority of the previous studies utilized pairwise, temporal synchronization-based FC. Recently, high-order FC (HOFC) methods were proposed with the idea of computing “correlation of correlations” to capture high-level, more complex associations among the brain regions. There are two types of HOFC. The first type is *topographical profile similarity-based HOFC* (*t*HOFC) and its variant, *associated HOFC* (*a*HOFC), for capturing different levels of HOFC. Instead of measuring the similarity of the original rs-fMRI signals with the traditional FC (low-order FC, or LOFC), tHOFC measures the similarity of LOFC profiles (i.e., a set of LOFC values between a region and all other regions) between each pair of brain regions. The second type is *dynamics-based HOFC* (*d*HOFC) which defines the quadruple relationship among every four brain regions by first calculating two pairwise dynamic LOFC “time series” and then measuring their temporal synchronization (i.e., temporal correlation of the LOFC fluctuations, not the BOLD fluctuations). Applications have shown the superiority of HOFC in both disease biomarker detection and individualized diagnosis than LOFC. However, no study has been carried out for the assessment of test-retest reliability of different HOFC metrics. In this paper, we systematically evaluate the reliability of the two types of HOFC methods using test-retest rs-fMRI data from 25 (12 females, age 24.48 ± 2.55 years) young healthy adults with seven repeated scans (with interval = 3–8 days). We found that all HOFC metrics have satisfactory reliability, specifically (1) fair-to-good for tHOFC and aHOFC, and (2) fair-to-moderate for dHOFC with relatively strong connectivity strength. We further give an in-depth analysis of the biological meanings of each HOFC metric and highlight their differences compared to the LOFC from the aspects of cross-level information exchanges, within-/between-network connectivity, and modulatory connectivity. In addition, how the dynamic analysis parameter (i.e., sliding window length) affects dHOFC reliability is also investigated. Our study reveals unique functional associations characterized by the HOFC metrics. Guidance and recommendations for future applications and clinical research using HOFC are provided. This study has made a further step toward unveiling more complex human brain connectome.

## Introduction

Functional connectivity (FC), as originally proposed as the temporal dependence between different spatially-distant brain regions (Friston et al., 1993), has become the major method to analyze resting-state functional magnetic resonance imaging (rs-fMRI) data (Biswal et al., 2010; Fox and Greicius, 2010; Van Dijk et al., 2010; Friston, 2011; Yeo et al., 2011; Fox et al., 2012). Except for the seed-based correlation that mainly focuses on voxel-wise massive one-to-one FC, the mostly adopted FC analysis strategy is pairwise correlation of region-averaged rs-fMRI signals for each pair of *N* brain regions, often resulting in an *N* × *N* FC matrix that represents whole-brain functional connectome. Various post-processing methods can be applied to these matrices to detect the potential connectivity biomarkers for brain diseases, including mass-univariate analyses that reveal group-level FC differences, or pattern cognition and individualized classification based on the features of all the FCs.

**Figure 1:**
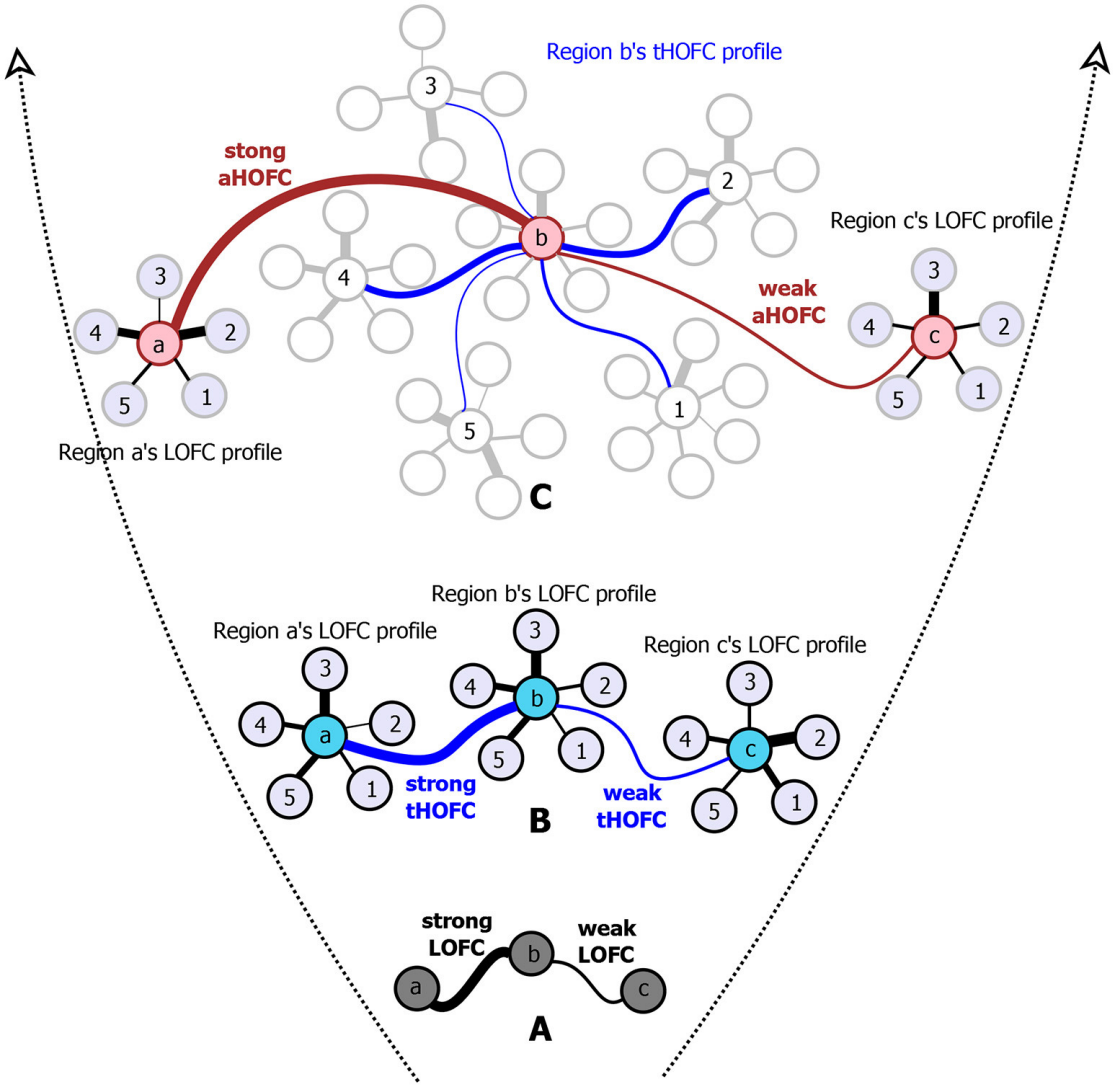
The diagram for illustrating the hierarchical definitions of LOFC **(A)**, tHOFC **(B)**, and aHOFC **(C)**. The original version of tHOFC is illustrated in the subplot **(B)**, and its variant measuring inter-level interactions, namely the associated HOFC (aHOFC), is illustrated in the subplot **(C)**. For simplicity, only three regions of interest (regions a, b, and c) are used to demonstrate the LOFC and the HOFC. For an illustration of the LOFC profiles of each region in **(B)**, only five other brain regions are used (regions 1–5) to calculate the LOFC strength with regions a–c. For each region's tHOFC profile, five of the regions 1–5's LOFC profiles are used for illustration, each of which has 4–6 regions connected. Different line widths indicate different connectivity strengths. For each type of connectivity metrics, we show both strong and weak connectivity strengths. The black curves indicate the LOFC, the blue curves represent the tHOFC, and the red curves depict the aHOFC.

However, such a one-to-one pairwise FC calculation has a well-known limitation since it reveals only simple temporal synchronization between two brain regions (Figure 1A). With simple FC, the high-level relationship among the brain regions may not be fully captured. To address this issue, we have proposed several metrics to capture high-level relationship based on “correlation's correlation,” namely high-order FC (HOFC), among the brain regions. There are two *major types* of HOFC. The first is calculated based on the topological architecture of the complex brain FC networks. As shown in Figure 1B, by extracting a regional one-to-all FC profile that constitutes a set of the FC strengths between one region to all other regions, we can characterize the FC topographical similarity for each pair of the brain regions by calculating a second round of correlation on these regional FC profiles (Zhang H. et al., 2016). This metric captures the high-level functional similarities between two brain regions beyond the traditional temporal synchronization based merely on the raw rs-fMRI signals. We have shown that, with such a “correlation of correlations” strategy, this HOFC metric reveals complementary information to the traditional FC for biomarker detection for brain disease (Zhang H. et al., 2016). From then on, we call the traditional FC as low-order FC (*LOFC*), and use *topographical profile similarity-based HOFC* (*tHOFC*) to name this topographical similarity-based HOFC method. If two regions have strong tHOFC, they have quite similar LOFC patterns to all the brain regions but they may have quite distinct rs-fMRI signals. Further comparison of tHOFC between the mild cognitive impairment (MCI) and the healthy elderly groups has unveiled novel potential biomarkers for early Alzheimer's diseases (AD) detection (Zhang H. et al., 2016). Later on, a variant of tHOFC, named as “associated HOFC (*aHOFC*),” was also proposed for further assessment of the resemblance between the topographical profile of LOFC and that of the tHOFC (Figure 1C), which indicates a modulation and inter-level functional association between the low- and high-level functional organizations. aHOFC has demonstrated its better performance than LOFC and even the tHOFC in MCI classification (Zhang et al., 2017). Of note, although both tHOFC and aHOFC measure high-level functional association, it is still the pairwise relationship characterized, similar to pairwise LOFC.

**Figure 2:**
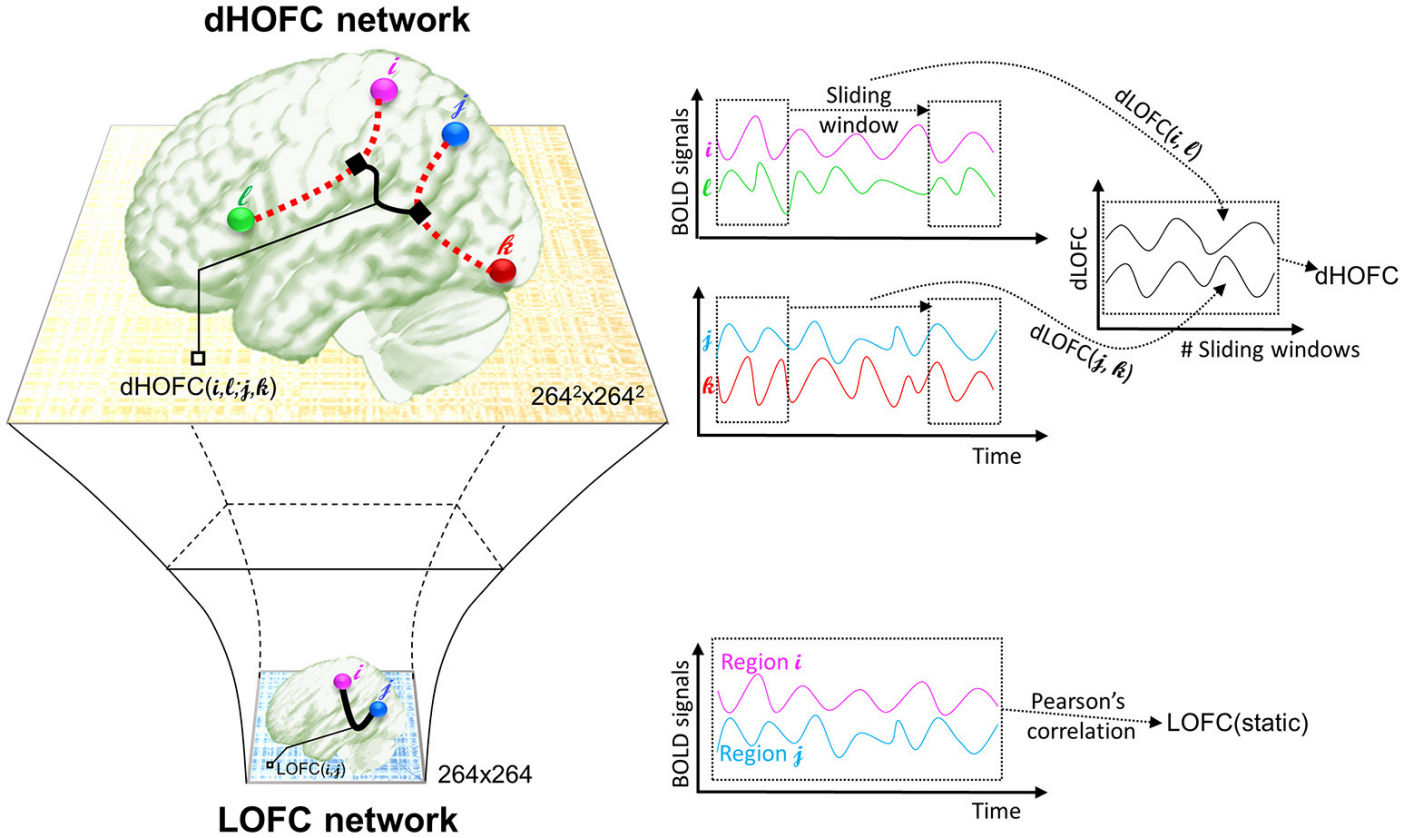
The calculation of dHOFC. This schematic plot shows how dHOFC is calculated and how the amount of information is increased from LOFC network to dHOFC network. Pairwise static LOFC only generates a 264 × 264 matrix, representing the static low-order brain functional network. By performing the sliding-window based dynamic LOFC (dLOFC) calculation for each pair of brain regions (i.e., *i* and *l*, and *j* and *k*, respectively), two dLOFC time series are generated. A further correlation of these two time series produces dHOFC among the four regions: *i, l, j*, and *k*. The information is geometrically increased in its amount, when using dHOFC matrix rather than LOFC to represent a brain network.

The *second* type of HOFC is based on a different interpretation of the “correlations' correlation” and can measure more complex relationship than a pairwise one. Rather than using the whole length of the rs-fMRI signals to obtain *static* LOFC, we use a brief segment of the data to conduct LOFC analysis for generating an instantaneous whole-brain LOFC network. By moving the window segment forward, a set of “dynamic” whole-brain LOFC is generated. For each pair of the brain regions, there is a *dynamic* LOFC time series reflecting the time-varying LOFC; it can be further correlated with the *dynamic* LOFC time series from another pair of brain regions, thus measuring high-level, quadruple interactions among four brain regions or two brain region pairs (Chen et al., 2016a). We call this as dynamics-based HOFC (*dHOFC*), which can be regarded as a “hyperlink” connecting two “hypernodes,” and each of the hypernodes represent a regular link between two brain regions (Figure 2). Since the dynamic LOFC may reflect adaptive and state-related temporary functional architecture, the dHOFC can measure the coherence of such processes, thus can reveal what LOFC cannot find. In addition, as shown in Figure 2, by calculating dHOFC on every quadruplet, we get a larger connectivity matrix of dHOFC compared with the small LOFC matrix. This indicates that we can use dHOFC to further construct more complex brain functional networks with more information introduced. This HOFC method has been successfully applied to early MCI detection (Chen et al., 2016a) and early AD detection (Chen et al., 2016b), as well as prediction of overall survival time of patients with brain gliomas (Liu et al., 2016), all with significantly better accuracy than LOFC.

Despite success in the abovementioned series of studies and the promising future of the HOFC applications, an essential question of how reliable and reproducible of such high-level FC metrics is still unanswered. Compared to the traditional LOFC with its test-retest reliability systematically assessed, which is fair-to-good when examined in both region- (Wang et al., 2011) and voxel-wise manners (Shehzad et al., 2009; Somandepalli et al., 2015), the state-of-the-art HOFC algorithms still lack comprehensive reliability assessment. Timely evaluation of HOFC reliability is crucial for their broader applications. Only with adequate reliability, we can then expect the detected HOFC biomarkers, or the HOFC-based disease detection, to be reproducible. Notably, the recent revisits of previously famous biomarker detection studies have found that those biomarkers could not be properly reproduced (Horrigan et al., 2017), which has been ringing a warning bell to the field and further increases the urgency of HOFC reliability study. In this paper, we will systematically evaluate the test-retest reliability of both topographical similarity-based HOFC (tHOFC and aHOFC) and dynamics-based HOFC (dHOFC) at the single connection level using repeated rs-fMRI scans. Note that good test-retest reliability of HOFC will indicate that the estimated HOFC from a subject based on one rs-fMRI session can be largely replicated based on the data of the same subject but from another rs-fMRI session. In addition, if a method or metric is proven to be test-retest reliable, its result could be more robust to noise, thus can be more easily to be reproduced by other researchers. Another aim of this HOFC test-retest reliability study is to investigate the underlying neurobiological meaning of the HOFC metrics according to the reliability evaluation. Test-retest reliability and its difference for different connections are informative to let us draw conclusions, especially on differentiating the noise from the signal. For example, previous studies found that the noise-related component derived from independent component analysis (ICA) on the rs-fMRI have lower test-retest reliability than that of the biologically meaningful components representing brain functional networks (Zuo et al., 2010). Finally, as the HOFC is still a new concept to the field, a timely test-retest reliability assessment will provide guidelines to further studies to prevent from unreliable results being misinterpreted.

We hypothesize that all the HOFC metrics (tHOFC, aHOFC, and dHOFC) have at least fair test-retest reliability, which means the major pattern of the HOFC can be largely reproduced based on a repeated rs-fMRI scan, because these metrics were proposed to reflect stable and biologically meaningful brain functional organizations that could thus be consistent. Different from tHOFC and aHOFC, dHOFC is based on dynamic LOFC which captures transient brain states. Although such dynamic LOFC could be different at a different time (such as different rs-fMRI scans), we think that the second round of correlations based on the dynamic LOFC time series could produce stable dHOFC that may reflect higher-level brain functional organization (i.e., synchronized brain state transition). Therefore, we also proposed that the dHOFC is considerably test-retest reliable. As an important influencing factor, whether different parameter settings such as different sliding window lengths could affect the dHOFC reliability will be also investigated. Based on the reliability results, practical guidelines and recommendations are provided for future studies.

## Materials and methods

### Data

We adopted a publicly available test-retest data (http://dx.doi.org/10.15387/fcp_indi.corr.hnu1) as part of the Consortium for Reliability and Reproducibility (CoRR) (Zuo et al., 2014). This dataset includes 30 healthy adults (aged 20–30 years old, 15 females) with 10 repeated rs-fMRI scans (sessions) within 1 month. The 10-session rs-fMRI scans are essential for more accurate reliability estimation because it constitutes adequate samples at *both* the group (# of repeated scans) *and* individual (# of subjects) levels; however, many previous test-retest reliability studies only used two sessions (Zhang et al., 2011a,b). Based on this dataset, intra-class correlation (ICC) for test-retest reliability evaluation can be more accurately estimated based on multiple repeated scans. Another advantage of this dataset is that the whole period of data collection was completed within 1 month, with each rs-fMRI session separated by 3–4 days. This reduces the potential inference of other longitudinal factors to the reliability estimation, such as development, plasticity, etc. This study was carried out in accordance with the recommendations of the ethics committee of the Center for Cognition and Brain Disorders at Hangzhou Normal University. All subjects gave written informed consent in accordance with the Declaration of Helsinki. The protocol was approved by the ethics committee of the Center for Cognition and Brain Disorders at Hangzhou Normal University.

The data was acquired by a GE MR750 3.0 Tesla MRI scanner, including both a T1-weighted image (used for rs-fMRI registration) and an rs-fMRI (echo-planar imaging, TR/TE = 2,000/30 ms, voxel size = 3.4 × 3.4 × 3.4 mm^3^, slice number = 43, matrix size = 64 × 64 × 64, 10 min, 300 time points). During rs-fMRI, all subjects stared at a fixation point on the screen without falling asleep. For detailed data information, please refer to the data release and CoRR websites.

### Data preprocessing

The rs-fMRI preprocessing was carried out based on DPARSF v2.3 (Yan and Zang, 2010) with routine procedures following the previous studies (Mao et al., 2015; Yu et al., 2017). It includes: (1) removing the first 5 time points, (2) slice timing correction, (3) head motion correction, (4) unified segmentation of the T1-weighted image after it was aligned to the rs-fMRI data, (5) warping the rs-fMRI data based on the deformation field produced by the previous step to the Montreal Neurological Institute (MNI) standard space, (6) spatially smoothing with a 6-mm Full Width at Half Maximum (FWHM) isotropic Gaussian kernel, (7) band-pass filtering (0.01–0.1 Hz), and 8) regressing out covariate signals including the first- and second-order polynomial functions, averaged signals from the white matter and cerebrospinal fluid (CSF), as well as the *Friston 24*-parameter head motion curves. Similar to our previous works (Chen et al., 2016a), we did not conduct data scrubbing to remove the data with larger frame-wise head motion. Although this step could further reduce head motion effect to LOFC analysis (Power et al., 2014), scrubbing itself will interrupt the temporal structure of the data and probably introduce artifacts into the dynamic LOFC analysis (Hutchison et al., 2013) before the dHOFC calculation. Instead, we used a stringent head motion exclusion criterion. That is, the subject with head motion larger than 1.5 mm or 1.5° in *any* rs-fMRI session was discarded. The rs-fMRI sessions with too many (>3) subjects discarded were not used for the test-retest reliability estimation. Therefore, sessions #2, #6, and #10 were discarded. Only 7 rs-fMRI sessions and 25 subjects (13 males, 12 females, age 24.48 ± 2.55 years old, ranging from 20 to 30 years old) were finally chosen for the following analysis. We also calculated the percentage of the rs-fMRI frames with excessive (>0.5) frame-wise displacement based on Power et al.'s method (Power et al., 2014) for each subject and each session; all the remained subjects have < 5% (i.e., 14) frames with excessive micro-head motion. In addition, we further test whether data scrubbing will affect the LOFC, tHOFC, and aHOFC estimation by conducting the similar analysis based on the scrubbed data; as we anticipated, the reliability did not change significantly.

### LOFC: temporal synchronization of rs-fMRI signals

We first calculate the traditional FC (i.e., LOFC) based on the pair-wise temporal correlation of the preprocessed rs-fMRI signals for each of two brain regions using Pearson's correlation. Letting *x*_*i*_(*t*) and *x*_*j*_(*t*) represent the rs-fMRI signals for two brain regions *i* and *j* at time point *t* (*t* = 1, …, *T*), the LOFC_*ij*_ can be defined as

$${\text{LOFC}}_{ij}=\frac{\sum_{t=1}^{T}\left(x_{i}\left(t\right)-\bar{x_{i}}\right)\left(x_{j}\left(t\right)-\bar{x_{j}}\right)}{\sqrt{\sum_{t=1}^{T}{\left(x_{i}\left(t\right)-\bar{x_{i}}\right)}^{2}{\sum_{t=1}^{T}\left(x_{i}\left(t\right)-\bar{x_{i}}\right)}^{2}}}$$

where $\bar{x_{i}}$ is the mean of the rs-fMRI signals at region *i*. A 264-region brain atlas (Power et al., 2013) was used to parcellate the brain; each region of interest (ROI) was represented by a sphere with 5-mm radius. The mean rs-fMRI signals from each ROI were extracted. LOFC matrix with the size of 264 × 264 was calculated for each subject for each session, which served as a baseline for comparison with the topographical similarity-based HOFC methods (tHOFC/aHOFC).

### tHOFC/aHOFC: similarity of topographical connectivity profiles

tHOFC and aHOFC calculations are straightforward without free parameters to be estimated, both of which characterize the relationship between two brain regions. However, they characterize different pairwise relationship from that of the LOFC due to the difference in the input “signals” (for LOFC calculation, the input signals are the rs-fMRI time series; but, for tHOFC, they are regional LOFC profile). Different input signals may cause prominent difference between LOFC and tHOFC (or aHOFC) between the same two brain regions. In fact, we have found that two brain regions with little temporal synchronization (indicating weak LOFC) have highly similar topographical LOFC profiles (suggesting strong tHOFC).

Specifically, tHOFC was calculated by column-wise correlation for each pair of the columns (with each column representing the LOFC profile of each brain region) from the 264 × 264 LOFC matrix. Letting LOFC_*i*_ represents the LOFC profile for region *i*, and LOFC_*i*._ = {LOFC_ik|k ∈ **R**,k ≠ i_} (where **R** is the set of all brain regions), the tHOFC_*ij*_ can be defined as,

$${\text{tHOFC}}_{ij}\text{​}=\text{​}\frac{\sum_{k}\left({\text{LOFC}}_{ik}-{\bar{\text{LOFC}}}_{i}.\right)\left({\text{LOFC}}_{jk}-\bar{{\text{LOFC}}_{j}.}\right)}{\sqrt{{\sum_{k}\left({\text{LOFC}}_{ik}-\bar{{\text{LOFC}}_{i}.}\right)}^{2}}\sqrt{{\sum_{k}\left({\text{LOFC}}_{jk}-\bar{{\text{LOFC}}_{i}}.\right)}^{2}}}$$

where *k* ∈ **R**, *k* ≠ *i, j*. Before such correlation, all the LOFC values were transformed to *z*-scores using Fisher's *r*-to-*z* transformation to satisfy the hypothesis of the second round of Pearson's correlation. Of note, self-connections of the two regions were excluded, i.e., the LOFC profile of each region is a 262-length vector (262 = 264–2).

The aHOFC is defined further based on the topographical profiles of the tHOFC. It measures the similarity between the *LOFC* topographical profiles and the *tHOFC* topographical profiles. Each brain region, when viewed from different levels, could interact with all other regions in both low-level (i.e., LOFC) and high-level (i.e., HOFC) manners. The aHOFC focuses on such a modulatory association between the two levels. During the aHOFC calculation, both LOFC and tHOFC profiles were first transformed into *z*-scores; the self-connections were ignored, similar to the calculation of tHOFC. Similarly, we use *tHOFC*_*i*_. to represent the tHOFC profile for region *i*, where HOFC_*i*._ = {HOFC_*ik*|*k* ∈ **R**,*k* ≠ *i*_}. The Pearson's correlation between any tHOFC_*i*_ and any LOFC_*j*_ defines aHOFC_*ij*_:

$$\begin{array}{l} {\text{aHOFC}}_{ij}=\\ \frac{\sum_{k}\left({\text{tHOFC}}_{ik}-\bar{{\text{tHOFC}}_{i.}}\right)\left({\text{LOFC}}_{jk}-\bar{{\text{LOFC}}_{j}.}\right)}{\sqrt{{\sum_{k}\left({\text{tHOFC}}_{ik}-\bar{{\text{tHOFC}}_{i}}\right)}^{2}}\sqrt{{\sum_{k}\left({\text{LOFC}}_{jk}-\bar{{\text{LOFC}}_{j.}}\right)}^{2}}} \end{array}$$

where *k* ∈ **R**, *k* ≠ *i, j*. The motivation of aHOFC is that, we think there are not only low-level and high-level FCs in the brain, but also inter-level interactions between LOFC and tHOFC connecting the two levels of FCs, similar to the common observations in many other biological networks, e.g., hierarchical organization and self-resemblance across multiple spatial scales (Guimera et al., 2003). Supposing that, in the human brain, the LOFC may collect and process information and the tHOFC may abstract information via the hierarchy (i.e., correlation's correlation), the possible functions of such inter-level connections could be (1) to facilitate the two levels of information talking to each other, (2) to let the low-level information guide high-level abstraction, and (3) to change the way of low-level information collection for a better high-level information integration. In addition, from robust system point of view, a network or complex biological system could be less fragile and more resilient to the targeted pathological attacks if it has inter-level connections. Taking brain psychophysiological interaction modeling as an example, high-level preset of a psychological status (e.g., attention level) may change sensory information collection, processing, and synthesis. All the evidence together suggests the existence of such an inter-level connection. We have applied the aHOFC to early detection of AD; compared with LOFC, using aHOFC as features not only improved the classification accuracy (Zhang et al., 2017) but also identified different discriminative features as potential AD biomarkers.

Of note, by definition, *tHOFC*_*i*_ and *LOFC*_*i*_ could be different, and thus it is also possible to calculate “self-associated HOFC” or *aHOFC*_*ii*_. Similarly, *aHOFC*_*ij*_ is not necessary to equal to *aHOFC*_*ji*_. Different from the previous study (Zhang et al., 2017), where the finally obtained aHOFC matrices were converted to be symmetric by adding each subject's dHOFC matrix with its transpose and dividing the result by two, we did not force the aHOFC matrices derived in this study to be symmetric as our purpose was to assess the aHOFC's reliability rather than to construct undirected aHOFC networks and make certain neurobiological conclusions. However, to make the mean connectivity matrices comparable among LOFC, tHOFC and aHOFC, we changed the diagonal values in the finally obtained aHOFC matrix to be zeros, i.e., in this study we did not count for the self-associated HOFC. In the future, we should further use the directed aHOFC network with non-zero self-associated HOFC as defined by an asymmetric aHOFC matrix and apply directed network analysis methods on the aHOFC network to reveal more information. Figure 1 summarize all the three pairwise FC metrics. See the first three columns of the Table 1 for the summarized differences among these three FC metrics.

**Table 1:** Differences among LOFC and various HOFC metrics.

	**Input**	**Output**	**Test-retest reliability**
LOFC	BOLD signals	Temporal synchronization, functional coherence	Fair-to-good; nearly all connections have fair or better reliability. Within-network connections have better reliability; high-level cognitive function-related connections have better reliability.
tHOFC	Regional LOFC topographical profiles	To what extent two regions share similar LOFC topographical profiles	Fair-to-good; similar to LOFC reliability, but with reduced reliability at within-network connections. Better reliability at inter-network connections (esp. between high-level cognition and primary regions).
aHOFC	Both regional LOFC and regional tHOFC topographical profiles	To what extent topographical LOFC modulates topographical tHOFC	Fair-to-good; similar to LOFC reliability, but with further reduced reliability at within-network connections. Better reliability at inter-network connections.
dHOFC	Dynamic, time varying LOFC time series between two brain regions	Temporal synchronization of two time-varying LOFC time series among four brain regions	Fewer connections have fair or better reliability. Strong (within-network and modulatory) connections have fair-to-moderate reliability. Between-network connections have poor reliability. Shorter window length produces better reliability.

*BOLD, Blood-oxygen-level dependent; LOFC, low-order functional connectivity; tHOFC, topographical similarity-based high-order functional connectivity; aHOFC, associated HOFC; dHOFC, dynamics-based HOFC*.

### dHOFC: correlation between pairwise LOFC dynamics

The calculation of dHOFC is quite different from that of the topographical similarity-based HOFC metrics (tHOFC/dHOFC), as the tHOFC/aHOFC measures static connectivity but dHOFC is calculated based on dynamic, time-varying LOFC profiles. As for the network topology, dHOFC also differs from tHOFC and aHOFC. As shown in Figure 2 and summarized in the first three columns of Table 1, for dHOFC calculation, dynamic LOFC for each pair of the brain regions was first calculated using a widely adopted sliding-window strategy (i.e., with window length ω = 30 time points or 60 s, step size = 1 time point or 2 s); then two dynamic LOFC time series (involving four regions) were correlated using Pearson's correlation to produce dHOFC between one region pair to another region pair. Letting dLOFC(τ) represent the dynamic LOFC strength within a brief time window from τ to τ + ω − 1, and the dLOFC time series between region *i* and *l* can be characterized based on

$$\begin{array}{l} {\text{dLOFC}}_{il}\left(\tau \right)=\frac{\sum_{t=\tau }^{\tau +\omega -1}\left(x_{i}\left(t\right)-\bar{x_{i}^{\tau }}\right)\left(x_{l}\left(t\right)-\bar{x_{l}^{\tau }}\right)\;}{\sqrt{{\sum_{t=\tau }^{\tau +\omega -1}\left(x_{i}\left(t\right)-\bar{x_{i}^{\tau }}\right)}^{2}}\sqrt{{\sum_{t=\tau }^{\tau +\omega -1}\left(x_{l}\left(t\right)-\bar{x_{l}^{\tau }}\right)}^{2}}}\\ \;\left(\tau =1,\;\ldots ,T-\omega +1;i,l\in R,\;i\neq l\right) \end{array}$$

where $\bar{x_{i}^{\tau }}$ represents the mean value of such a brief segment of the rs-fMRI signal starting from τ. Similarly, we can define the dLOFC time series between regions *j* and *k* as dLOFC_*jk*_(τ) (τ = 1, …, *T* − ω + 1; *j, k* ∈ **R**, *j* ≠ *k*). The further Pearson's correlation between the two dLOFC time series defines dHOFC between region pairs *i – l* and *j – k* based on

$${\text{dHOFC}}_{il,jk}=\frac{\sum_{\tau =1}^{T-\omega +1}\left({\text{dLOFC}}_{\text{il}}\left(\tau \right)-\bar{{\text{dLOFC}}_{\text{il}}}\right)\left({\text{dLOFC}}_{\text{jk}}\left(\tau \right)-\bar{{\text{dLOFC}}_{\text{jk}}}\right)\;}{\sqrt{{\sum_{\tau =1}^{T-\omega +1}\left({\text{dLOFC}}_{\text{il}}\left(\tau \right)-\bar{{\text{dLOFC}}_{\text{il}}}\right)}^{2}}\sqrt{{\sum_{\tau =1}^{T-\omega +1}\left({\text{dLOFC}}_{\text{jk}}\left(\tau \right)-\bar{{\text{dLOFC}}_{\text{jk}}}\right)}^{2}}}$$

where $\bar{\text{dLOF}}{\text{C}}_{il}$ indicates the mean value of the *dLOFC*_*il*_ time series along the whole time. Based on the combination theory, a 264 × 264 LOFC matrix has 264 × (264–1)/2 = 34716 unique region pairs; thus a complete dHOFC network will have 34716 × 34716 in size and over 600 million unique four-region combinations. This will increase the amount of connectomic information and may reveal novel information that cannot be discovered by LOFC/tHOFC/dHOFC. For more details, please see the previous paper (Chen et al., 2016a).

**Figure 3:**
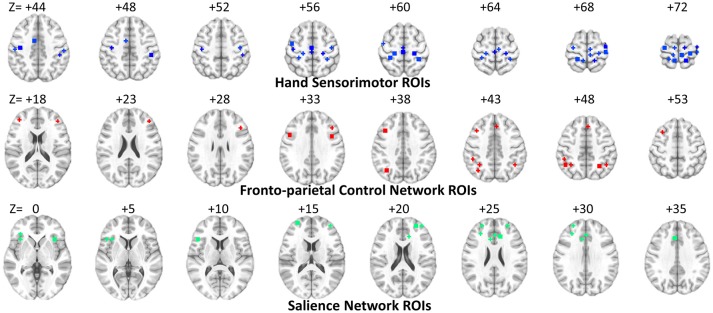
ROI locations for three functional networks used for dHOFC calculation. The underlying brain image is the ICBM152 template. Left is right and right is left.

In the previous classification-orientated studies, to avoid the curse of dimensionality, dHOFC matrix dimension was further reduced based on hierarchical clustering, which generates relatively fewer clusters by grouping similarly co-varied dynamic LOFC time series together. By doing so, we can detect a few hundreds of the clusters and calculate dHOFC based on the clusters' centroids (Chen et al., 2016a). In the current reliability study, it is not necessary to conduct such a clustering analysis because we are focusing only on the reliability of the dHOFC links, while clustering itself is irrelevant to such a goal and will unnecessarily introduce an additional parameter (i.e., the total number of clusters) which could complicate the current study. Therefore, in this paper, we chose a few ROIs from the total 264 of them to generate a relatively smaller and more interpretable dHOFC network. Specifically, we chose 26 ROIs from the hand-associated sensorimotor areas for investigation of the dHOFC in the primary functional system; we also chose 17 ROIs from the fronto-parietal task control network (FPN) and 15 from the salience network (SN) to investigate dHOFC in the high-level cognitive function-related brain systems. See Figure 3 and Table 2 for the details the ROI definitions. Therefore, we separately generated a dHOFC matrix for the primary areas (with the size of 325 × 325, where 325 = 26 × 25/2) and another dHOFC matrix for two high-level functional areas (with the size of 496 × 496, where 496 = 32 × 31/2, since there are totally 32 high-level function-related ROIs, 32 = 17 + 15). Of note, this is the first paper to systemically investigate the possible neurobiological correlation of the dHOFC in the specific functional systems.

**Table 2:** ROI definitions for dHOFC calculation.

**#**	**Orig #**	***x***	***y***	***z***	**Suggested system**	**#**	**Orig #**	***x***	***y***	***z***	**Suggested system**
1	16	10	−2	45	Sensorimotor Hand	1	186	47	10	33	Fronto-parietal Control
2	17	−7	−21	65	Sensorimotor Hand	2	187	−41	6	33	Fronto-parietal Control
3	18	−7	−33	72	Sensorimotor Hand	3	188	−42	38	21	Fronto-parietal Control
4	19	13	−33	75	Sensorimotor Hand	4	189	38	43	15	Fronto-parietal Control
5	20	−54	−23	43	Sensorimotor Hand	5	190	49	−42	45	Fronto-parietal Control
6	21	29	−17	71	Sensorimotor Hand	6	191	−28	−58	48	Fronto-parietal Control
7	22	10	−46	73	Sensorimotor Hand	7	192	44	−53	47	Fronto-parietal Control
8	23	−23	−30	72	Sensorimotor Hand	8	193	32	14	56	Fronto-parietal Control
9	24	−40	−19	54	Sensorimotor Hand	9	194	37	−65	40	Fronto-parietal Control
10	25	29	−39	59	Sensorimotor Hand	10	195	−42	−55	45	Fronto-parietal Control
11	26	50	−20	42	Sensorimotor Hand	11	196	40	18	40	Fronto-parietal Control
12	27	−38	−27	69	Sensorimotor Hand	12	197	−34	55	4	Fronto-parietal Control
13	28	20	−29	60	Sensorimotor Hand	13	198	−42	45	−2	Fronto-parietal Control
14	29	44	−8	57	Sensorimotor Hand	14	199	33	−53	44	Fronto-parietal Control
15	30	−29	−43	61	Sensorimotor Hand	15	200	43	49	−2	Fronto-parietal Control
16	31	10	−17	74	Sensorimotor Hand	16	201	−42	25	30	Fronto-parietal Control
17	32	22	−42	69	Sensorimotor Hand	17	202	−3	26	44	Fronto-parietal Control
18	33	−45	−32	47	Sensorimotor Hand	18	206	31	33	26	Salience Network
19	34	−21	−31	61	Sensorimotor Hand	19	207	48	22	10	Salience Network
20	35	−13	−17	75	Sensorimotor Hand	20	208	−35	20	0	Salience Network
21	36	42	−20	55	Sensorimotor Hand	21	209	36	22	3	Salience Network
22	37	−38	−15	69	Sensorimotor Hand	22	210	37	32	−2	Salience Network
23	38	−16	−46	73	Sensorimotor Hand	23	211	34	16	−8	Salience Network
24	39	2	−28	60	Sensorimotor Hand	24	212	−11	26	25	Salience Network
25	40	3	−17	58	Sensorimotor Hand	25	213	−1	15	44	Salience Network
26	41	38	−17	45	Sensorimotor Hand	26	214	−28	52	21	Salience Network
						27	215	0	30	27	Salience Network
						28	216	5	23	37	Salience Network
						29	217	10	22	27	Salience Network
						30	218	31	56	14	Salience Network
						31	219	26	50	27	Salience Network
						32	220	−39	51	17	Salience Network

*Orig #, Original ROI index in the 264 brain region atlas. x, y, z, coordinates of each ROI's center in the MNI space. Suggested system: the functional system suggested by the atlas. We deleted 3 ROIs in the salience network, 8 ROIs in the fronto-parietal control network, and 3 hand sensorimotor ROIs since their belongingness to the suggested functional systems is less replicable across different data sets as suggested by Power et al. (2013)*.

Different window length could affect the accuracy of dynamic LOFC (Hutchison et al., 2013); (Leonardi and Van De Ville, 2015; Zalesky and Breakspear, 2015), thus may further affect dHOFC and its reliability. Therefore, we further examined the relationship between the window length and the test-retest reliability of the dHOFC within the hand sensorimotor areas. The above calculation was repeated with different window length settings (i.e., 20, 40, and 50 time points, corresponding to 40, 80, and 100 s, as TR = 2 s). Of note, the window length of 20 and 30 time points are within the recommended range (30–60 s) from previous studies (Zalesky and Breakspear, 2015), while the larger values (i.e., 80–100 s) are also used in previous dynamic LOFC studies (Leonardi and Van De Ville, 2015).

In addition, according to the previous studies on dynamic LOFC, small correlation values from the dynamic analysis could probably be caused by random noise (Leonardi and Van De Ville, 2015), we think that the reliability of the dHOFC which has weak connectivity strength could also be mainly contributed by noise. To this end, we also used a dHOFC threshold of 0.36 as suggested by Leonardi and Van De Ville (2015) to further filter the dHOFC matrix. If there is a significant modular structure after thresholding, we may be able to draw a conclusion that, although weak dHOFC may be driven by noise, the relatively stronger dHOFC could be biologically meaningful. This is because that, if all dHOFC connections are dominated by noise, the thresholded dHOFC matrix will have a somewhat random spatial pattern rather than a structured one. Similar to the tHOFC and aHOFC, test-retest reliability was also calculated for the relatively strong dHOFC connectivities.

### Intra-class correlation for test-retest reliability evaluation

To investigate test-retest reliability of all types of HOFC connections, we utilized a commonly adopted index called ICC (Shrout and Fleiss, 1979). ICC is a method based on the one-way analysis of variance (ANOVA) which divides the total sum of variance across subjects and repeated rs-fMRI scans into two parts: between-subject ($\sigma_{b}^{2}$) and within-subject (or inter-session variance, $\sigma_{w}^{2}$) sum of variance. The theoretical definition of ICC is:

$$ICC=\;\frac{\sigma_{w}^{2}}{\sigma_{b}^{2}+\sigma_{w}^{2}};$$

but the estimation of the ICC based on real samples can be written by:

$$ICC=\;\frac{MS_{b}-MS_{w}}{MS_{b}+\left(k-1\right)\times MS_{w}},$$

where *MS*_*b*_ is the mean square of between-subject sum of variance, *MS*_*w*_ is the mean square of within-subject sum of variance, and *k* is the number of repeated rs-fMRI scans (here *k* = 7). ICC is conceptually positive between 0 (not reliable at all) and 1 (perfectly consistent between repeated measurements), but its estimation can be negative in a few cases. We put the negative ICC values to be zeros as always done by previous studies (Zhang et al., 2011a). Based on the value of ICC, reliability is usually categorized as poor (ICC = 0–0.2), fair (0.2–0.4), moderate (0.4–0.6), substantially good (0.6–0.8), and excellent (>0.8) (Landis and Koch, 1977; Chen et al., 2015).

We first calculated ICC for LOFC, tHOFC and aHOFC, as they are convenient to compare. We then calculate ICC for dHOFC in both primary functional systems (hand sensorimotor areas) and high-level cognition-related functional networks (FPN and SN), to compare the dHOFC in these primary and high-level functional systems.

## Results

### tHOFC and aHOFC have moderate-to-good test-retest reliability

**Figure 4:**
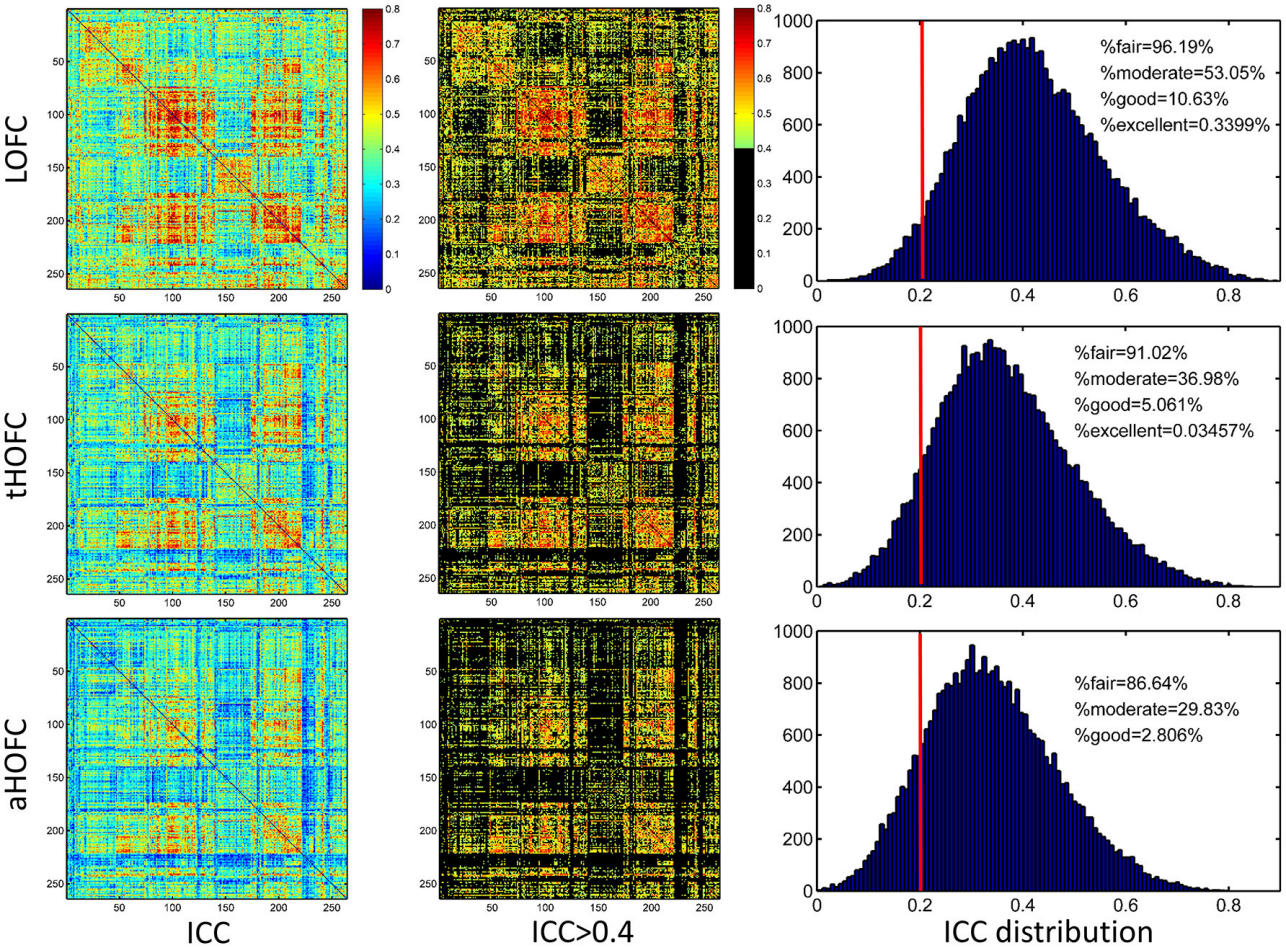
ICC value for each connection of LOFC, tHOFC, and aHOFC. The three rows show test-retest reliability, as assessed by ICC at each connection, for LOFC, tHOFC, and aHOFC, respectively. From left to right are the ICC matrix without thresholding, ICC matrix showing the connectivities with moderate or better reliability (thresholded by ICC > 0.4), and the ICC distribution for all connections. In the right of each row, the vertical red line indicates ICC = 0.2, above which are the connectivities with acceptable (fair or better) reliability; the percentage of the connection with fair, moderate, good and excellent reliability are also shown.

As shown in Figure 4, the test-retest reliability of the tHOFC is generally fair-to-moderate, although slightly lower than that of the LOFC. The test-retest reliability of the aHOFC is similar to that of the tHOFC, with slightly fewer connections having fair-to-moderate ICC. These results indicate that the tHOFC and aHOFC are still reliable metrics. An interesting finding is that the overall pattern of the reliable connections are quite consistent among the LOFC, tHOFC and dHOFC, all of which show prominently better reliability for the connections within default mode network (DMN), as well as those within the FPN and SN, respectively (see those major blocks in the main diagonal and off-diagonal of the Figure 4). In addition, we also notice that the off-diagonal connections among the DMN, FPN, and SN have also high reliability. All these high-reliability connections, although a little bit weakened, still exist for tHOFC and dHOFC.

### Links with increased reliability for tHOFC and aHOFC, compared with LOFC

**Figure 5:**
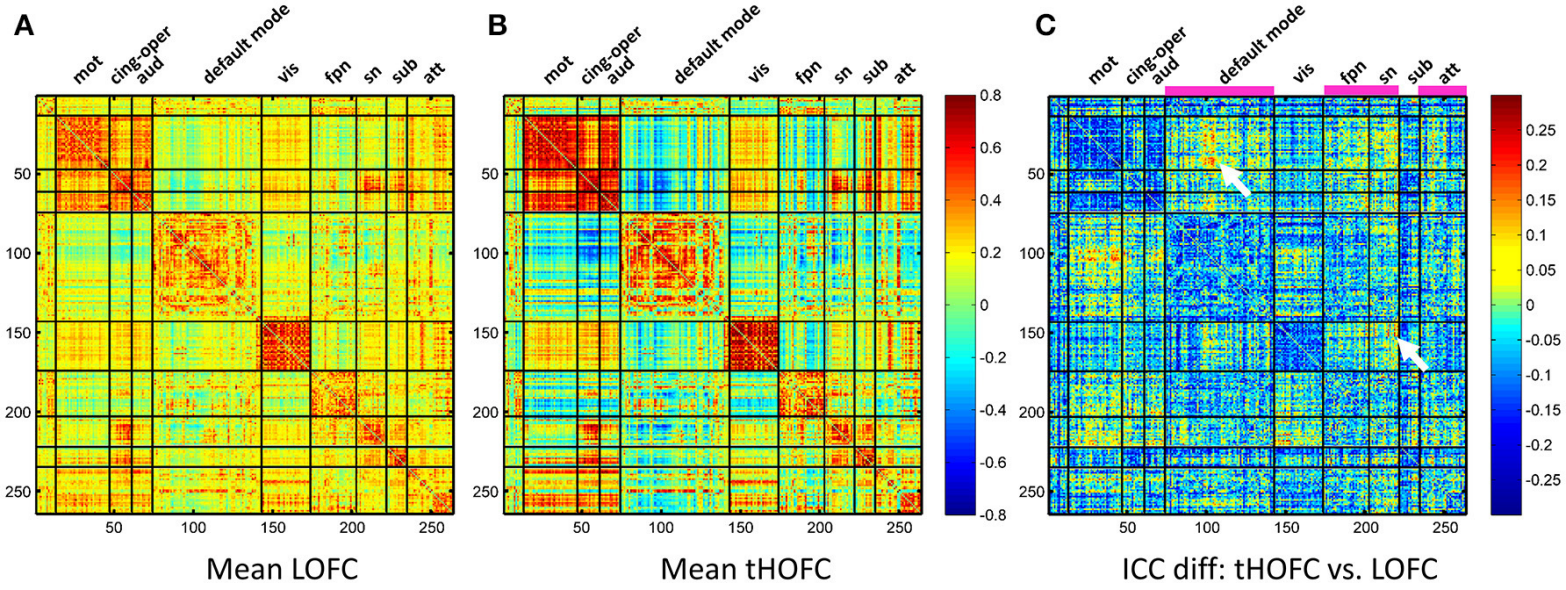
The test-retest reliability difference between tHOFC and LOFC. For better understanding which functional system contributes to such ICC increment, we also show the group-averaged LOFC in **(A)** and the group-averaged tHOFC in **(B)** across all subjects and all rs-fMRI sessions. Nine functional systems are shown, with higher intra-system connectivity and sparse inter-system connectivity. The tHOFC with increased reliability **(C)** are located mostly at *inter*-system links (as also highlighted by white arrows). The functional systems with many increased reliability are marked in **(C)** above the matrix and under the names of the functional systems. The abbreviations of the functional systems are: mot (sensorimotor), cing-oper (cingulo-opercular), aud (auditory), vis (visual), fpn (fronto-parietal task control network), sn (salience network), sub (subcortical regions), att (attention-related networks including the dorsal and ventral attentional systems).

**Figure 6:**
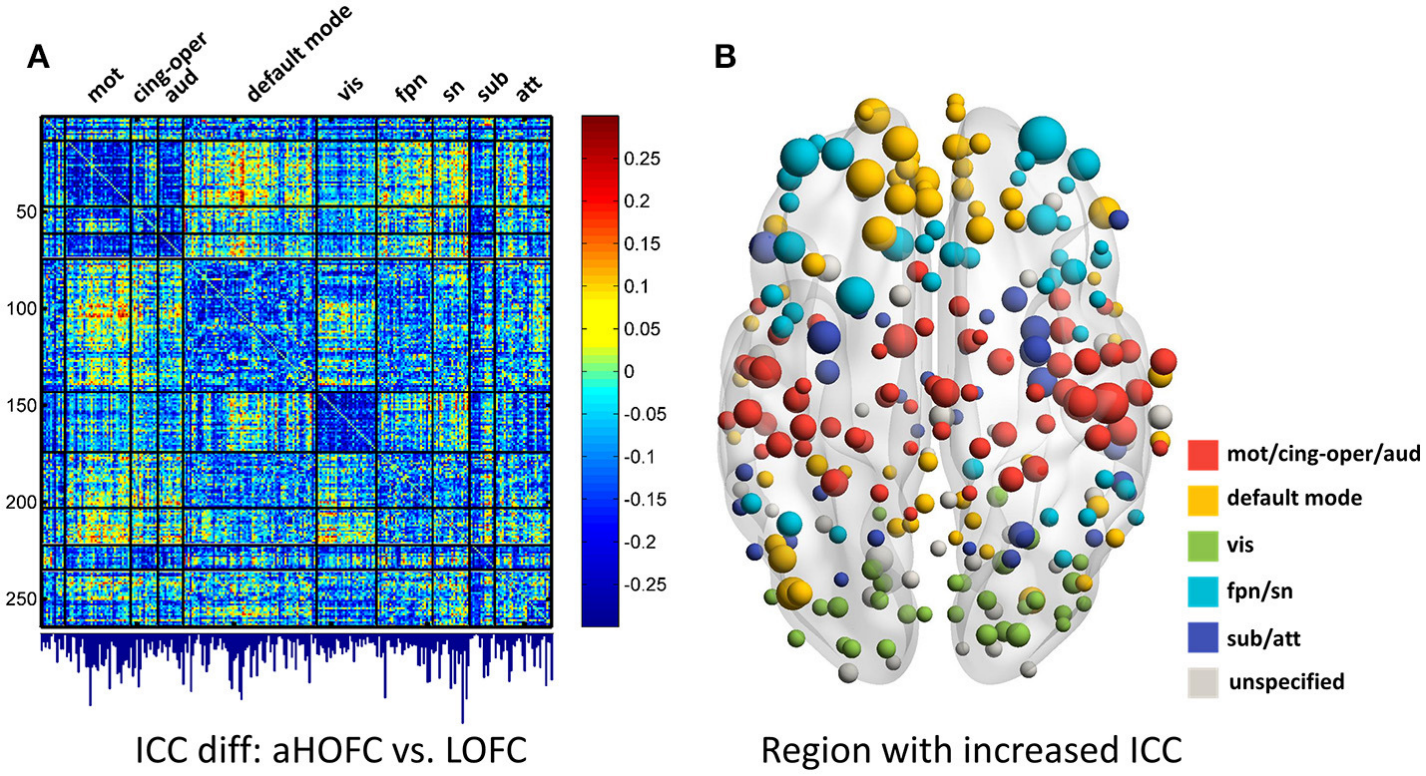
The test-retest reliability differences between aHOFC and LOFC. Subplot **(A)** shows the difference in ICC values between aHOFC and LOFC for all connections, with the quantitative measurement of ICC gain for each brain region (i.e., the sum of ICC increment across all the connections to each region) shown as a bar graph under the matrix. Such ICC increment is further visualized as the size of the node for all the brain regions in a brain surface **(B)**. Different colors indicate different functional systems.

In addition to the overall reduction of reliability for tHOFC/aHOFC compared with LOFC, we further found interesting increased reliability for several tHOFC (Figure 5) and aHOFC (Figure 6) links. Different from the reduced reliability for mainly intra-network strong connections (see Figures 5A,B for the block pattern), the links with increased reliability in tHOFC compared with LOFC are mainly located at the weak links that connect different systems. Specifically, we found that such links connect *high-level cognition-related* network (DMN, FPN, or SN) and *primary* function-related network (sensorimotor or visual network). For example, as indicated by white arrows in Figure 5C, the tHOFC links between the DMN and the hand sensorimotor regions, as well as those between the SN and visual areas, show great (by 0.2) increase in their ICC values. Notably, the group-averaged aHOFC matrix is quite similar to that for LOFC and tHOFC, with the strong aHOFC links mainly located within modules, and the weak aHOFC links between modules (result not shown). Similarily, aHOFC shows the similar result as the tHOFC for the links with increased ICC values, where such increase and reduction in the ICC values are even more prominent (Figure 6A).

We further show the specific brain regions with prominent reliability increment by comparing aHOFC with LOFC. To do this, for each brain region, we summarized the extent of ICC increment across all the aHOFC connections to this region with increased ICC. Different regions have various extent of reliability increment (see the bar plot under the matrix of Figure 6A). Such differences are further drawn in Figure 6B with different sizes of the nodes (with a bigger node indicating greater reliability increment for its aHOFC links). The brain regions with the greatest reliability increment are mainly distributed at the high-level cognitive function-related areas, such as the medial and lateral prefrontal cortices.

Taken together, our results show that both tHOFC and aHOFC have general moderate or better reliability, and that the tHOFC and aHOFC indeed capture novel (mostly high-level cognition-related) information as indirectly reflected by the higher reliability than LOFC.

### Strong dHOFC in the primary functional system has fair-to-moderate reliability

**Figure 7:**
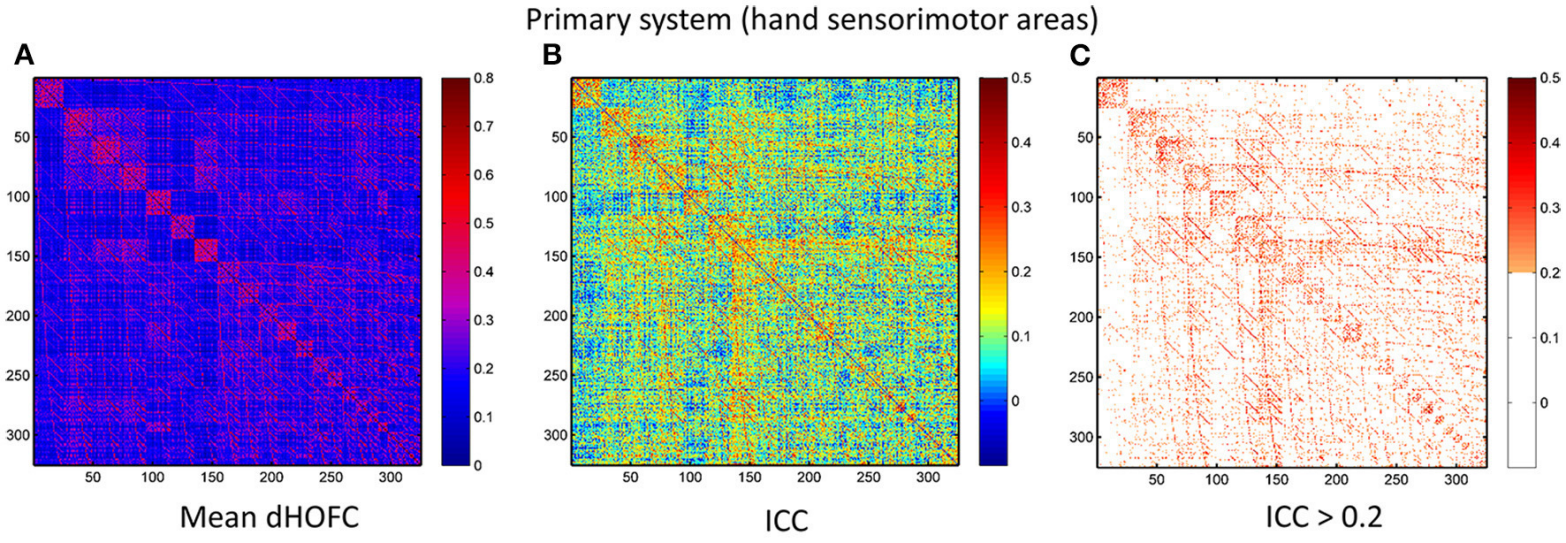
Test-retest reliability of dynamics-based HOFC (dHOFC) in the primary functional system. **(A)** Averaged dHOFC matrix across all subjects and all imaging sessions, showing the dHOFC strength for every possible high-order links; **(B)** ICC matrix, indicating test-retest reliability for all dHOFC links; **(C)** dHOFC links with fair or better (ICC > 0.2) test-retest reliability.

**Figure 8:**
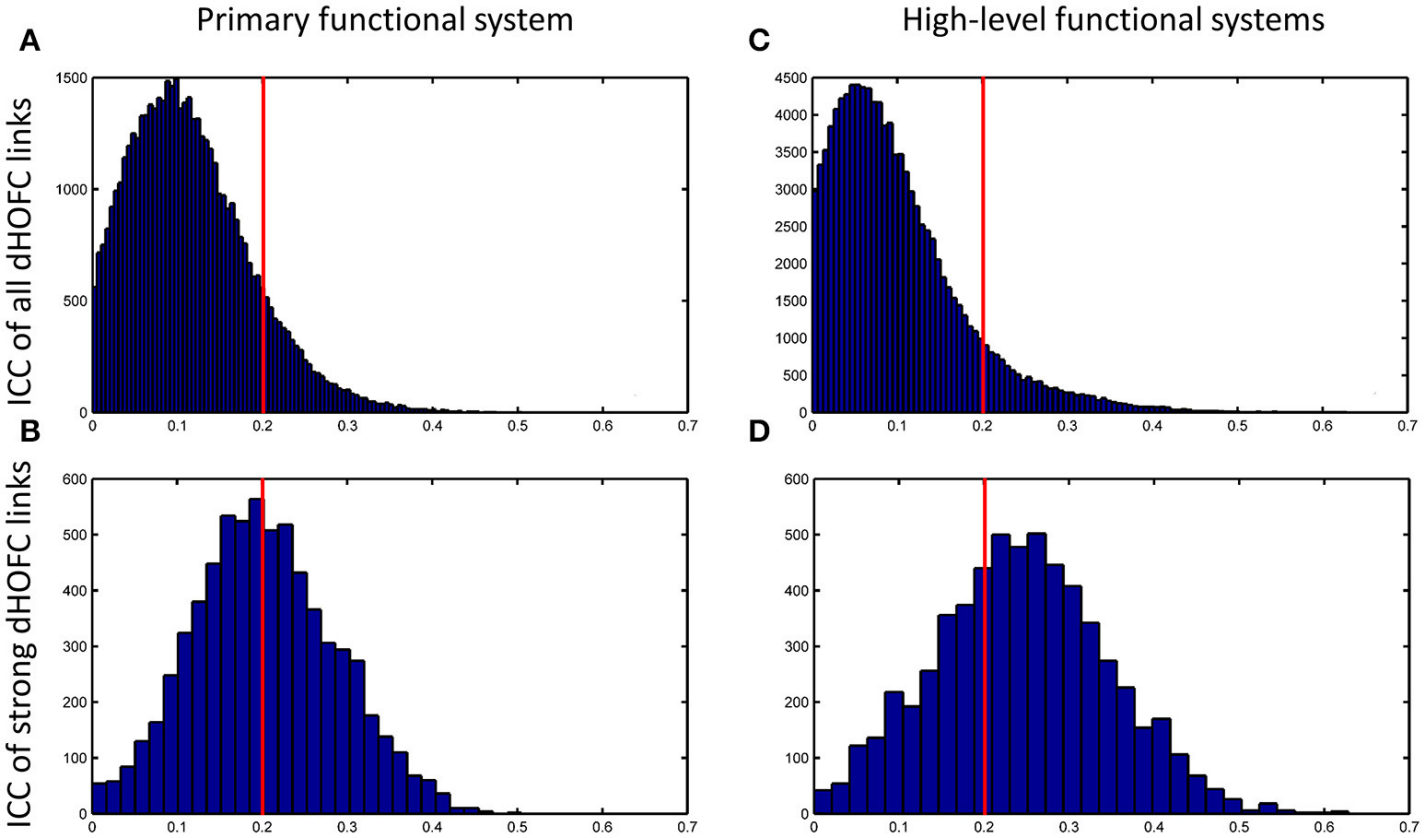
Distribution of test-retest reliability (ICC values) for the dHOFC links. **(A,B)** Results for the primary functional system; **(C,D)** results for the high-level functional systems. **(A,C)** show the distribution of the ICC values for all dHOFC links, while **(B,D)** show the distribution of the ICC values for the relatively strong (i.e., mean dHOFC > 0.36) dHOFC links. We selected the hand sensorimotor areas as an example of the primary functional system, and selected both fronto-parietal task control network and salience network as examples of the high-level cognition-related functional systems. Again, the red line indicates the same ICC threshold of 0.2; the bars on the right side of the red line are the numbers of dHOFC links with fair or better reliability.

**Figure 9:**
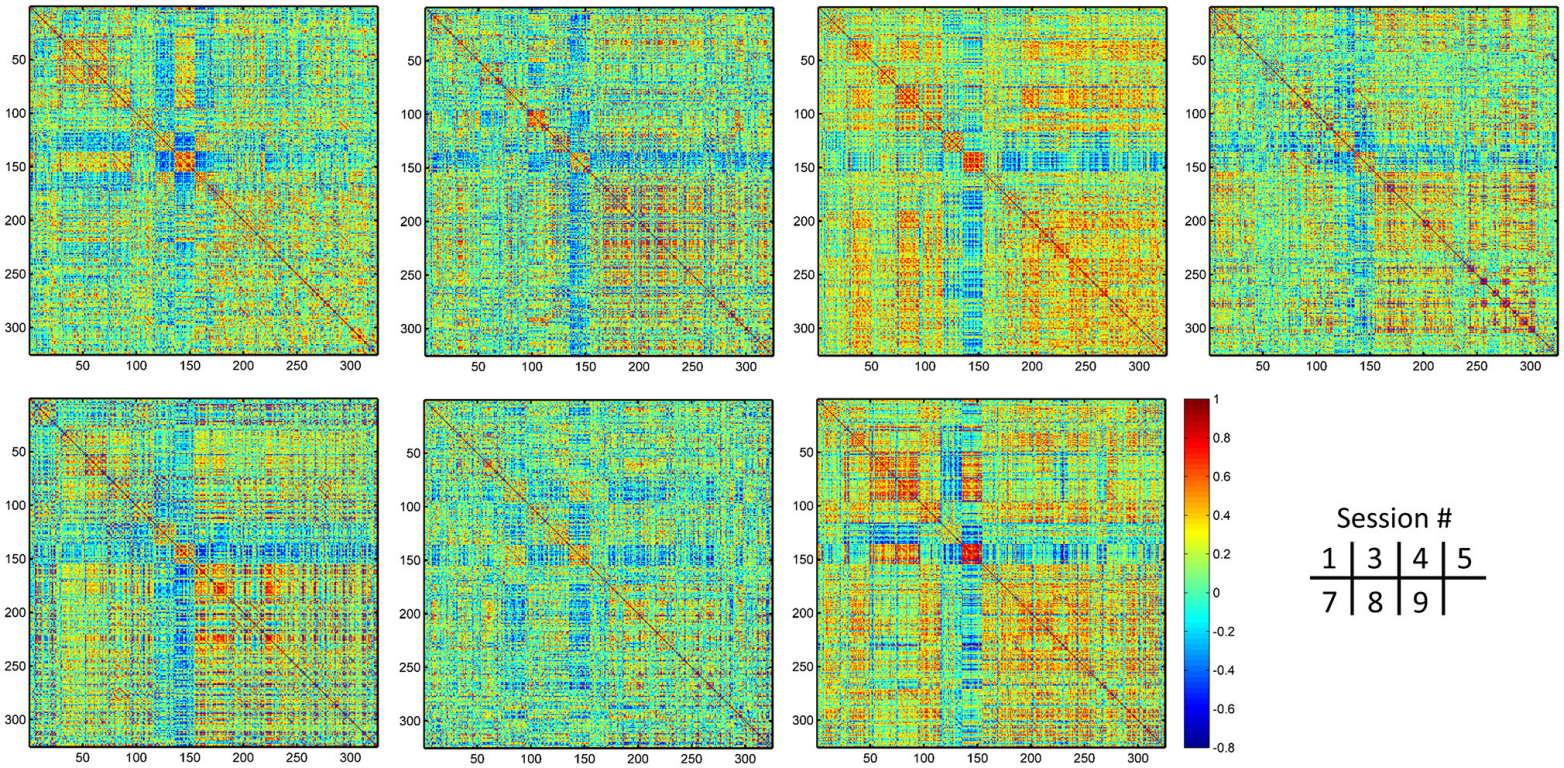
Individual dHOFC matrices for all 7 sessions. The dHOFC matrices for the hand sensorimotor network of a randomly selected subject (#9) are plotted.

The group-averaged dHOFC within 26 hand sensorimotor ROIs (one of the primary functional system) across all subjects and all sessions is represented by a larger matrix, which shows a significant structure with spatial sparsity (Figure 7A). For all the 325 × 324/2 = 52650 dHOFC hyperconnections (by treating the 325 region pairs as hypernodes), their test-retest reliability is shown as an ICC matrix with the same dimension (325 × 325) in Figure 7B, with its thresholded (ICC > 0.2) version (highlighting only the fairly or better reliable dHOFC) shown in Figure 7C. Although many dHOFC links have acceptable reliability as indicated by Figure 7C, specific amount of the dHOFC links with ICC > 0.2 is only 11.63% of all possible dHOFC links (Figure 8A). We have noted that the dHOFC links with higher reliability tend to be those with greater connectivity strength. If only counting for strong dHOFC (i.e., group mean dHOFC > 0.36), a half (49.5%) of such connections will have acceptable reliability (Figure 8B). Figure 9 shows the dHOFC matrix from a randomly selected subject from each of the seven rs-fMRI sessions. We can see that the overall individual dHOFC spatial patterns are consistent across different rs-fMRI sessions. However, there are significant block structures in the group averaged dHOFC matrix (Figure 7A) but it is less prominent at the individual-level (Figure 9). This difference could be due to the relatively high individual variability in many dHOFC links. While the group average could retain individually consistent dHOFC links, it also suppressed those with relatively high inter-subject variability, thus creating such prominent block structure in the mean dHOFC matrix.

### Strong dHOFC in the high-level functional systems has better reliability

**Figure 10:**
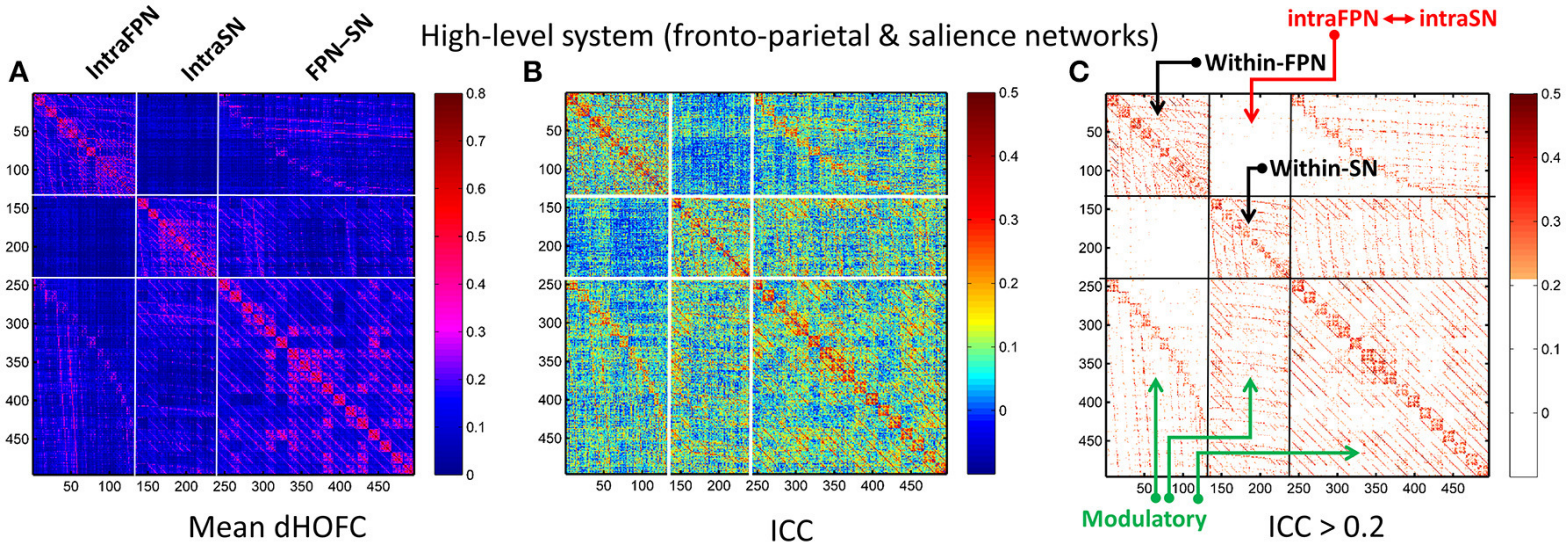
Test-retest reliability of dHOFC in the two high-level cognition-related functional systems. We selected the fronto-parietal task control network and salience network as examples of the high-level functional system. **(A)** Averaged dHOFC matrix; **(B)** ICC matrix for all dHOFC links; **(C)** ICC matrix for the dHOFC links with fair or better reliability (ICC > 0.2). The order of dHOFC links in the matrices is rearranged according to the types of the “hypernodes” (where a hypernode represents a dynamic link between two brain regions). If the hypernode consists of two brain regions that are both from the fronto-parietal task control network, we call it “*intraFPN”* hypernode and re-order them into the first 136 (136 = 17 × 16/2) columns of the dHOFC matrix. We further re-group the 105 (105 = 15 × 14/2) hypernodes which consist of two brain regions both from the salience network (*intraSN*) and put them after the intraFPN hypernodes. At last, we put all the remaining 255 (255 = 17 × 15) hypernodes (consisting of one region from FPN and the other from SN, thus called inter-network “*FPN-SN”* hypernodes) after the intraSN hypernodes. In this way, the dHOFC matrix is rearranged. According to different types of hypernodes, there are also three different types of (dHOFC) hyperlinks. Among them, the “*within-FPN”* (with both hypernodes being intraFPN nodes) and “*within-SN”* (with both hypernodes being intraSN nodes) are both indicated by black arrows; the between-network dHOFC hyperlinks (named here as “*intraFPN-intraSN*,” with one hypernode from intraFPN and another from intraSN) are indicated by the red arrows; and all the remaining dHOFC hyperlinks are named as “*modulatory”* dHOFC (with at least one hypernode belonging to the “*FPN-SN”* type) as indicated by the green arrows.

**Figure 11:**
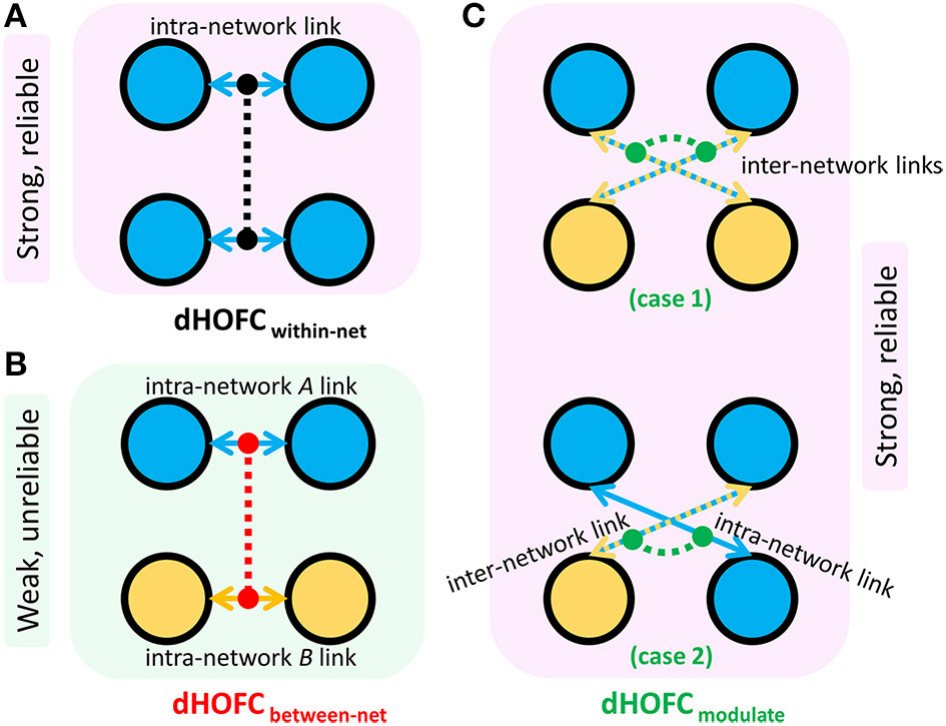
Three types of dHOFC and their overall connectivity strength and reliability. dHOFC_within-net_ is the within-network dHOFC, including within-FPN and within-SN hyperlinks **(A)**; dHOFC_between-net_ is the between-network dHOFC (including “intraFPN-intraSN” hyperlinks) **(B)**; dHOFC_modulate_ is the modulatory dHOFC links **(C)** which can be further categorized into two cases (case 1: both of the two hypernodes belong to the “*FPN-SN”* type; case 2: one of the two hypernodes belongs to the “*FPN-SN”* type while the other belonging to either *intraFPN* or *intraSN* type).

In addition to assessing the reliability for within-primary functional system dHOFC, we also investigated the reliability of high-level cognition-related dHOFC by calculating the dHOFC in the two typical high-order functional systems, i.e., the FPN and SN. Figure 10A shows the group-averaged dHOFC in these high-level systems, while Figure 10B shows their reliability. Since there are two functional systems involved, the dHOFC can be divided into three main types (see Figure 10C and also the summary in Figure 11) based on the functional system belongingness of the four brain regions that constitute a dHOFC hyperlink:

- *Within-network dHOFC*. For each dHOFC consisting of four ROIs, all ROIs belong to the same functional system. For example, a link between two intra-FPN ROIs (regarded as intra-FPN hypernode) has dHOFC with another link between two intra-FPN ROIs. In this case, both hypernodes are intra-FPN, thus we call this type of dHOFC links *within-FPN* dHOFC. Similarly, we can define *within-SN* dHOFC between two hypernodes that both constitute intra-SN ROIs. This type of the dHOFC characterizes within-network high-order relationship, which has moderate connectivity strength and acceptable reliability (see the first two big blocks in the main diagonal of the matrices in Figure 10).
- *Between-network dHOFC*. This type of dHOFC characterizes the high-order relationship *between* two intra-network links (or hypernodes) which belong to different functional networks. For example, a hypernode that connects two FPN ROIs has dHOFC with another hypernode that connects two SN ROIs (i.e., an “intraFPN-to-intraSN” hyperlink). This type of the dHOFC measures high-order functional association between two functional systems. Interestingly, such dHOFC are mostly weak in the connectivity strength and have overall poor reliability (Figures 10B,C).
- *Modulatory dHOFC*. This is a new type of connectivity that has not been defined in the previous studies. It contains two hypernodes, at least one of which contains an inter-network link. This type of the dHOFC constitutes the most part of the dHOFC matrix. There are two subtypes for the modulatory dHOFC. The first subtype consists of the dHOFC between one *inter-network* hypernode and one *intra-network* hypernode, e.g., the dHOFC between an intra-FPN hypernode and an FPN–SN link. The second subtype is that both of the hypernodes are the inter-network links. Both of these two cases are able to characterize high-order functional relationships manifesting as “one functional system *modulates* another.” Compared with the first two types of the dHOFC, the modulatory dHOFC show extensive connections (see the third main block in the main diagonal of Figure 10A) and acceptable reliability (Figure 10C).

As shown in Figure 10, the mean dHOFC strength matrix and the dHOFC reliability matrices have highly similar *structured* and *blocked* patterns. Please note that we did not re-arrange the columns and the rows of these matrices in a *post hoc* way (e.g., based on module detection using the dHOFC strength); instead, we just grouped the same type of the hypernodes (three types: intra-FPN, intra-SN, and FPN-to-SN) together before calculating dHOFC and re-arranging the columns and the rows of these matrices in an order of firstly intra-FPN, then intra-SN, and finally FPN-to-SN. Merely through this a priori grouping and rearranging could we reveal such an interesting structured and block-like pattern for both dHOFC strength and reliability.

**Figure 12:**
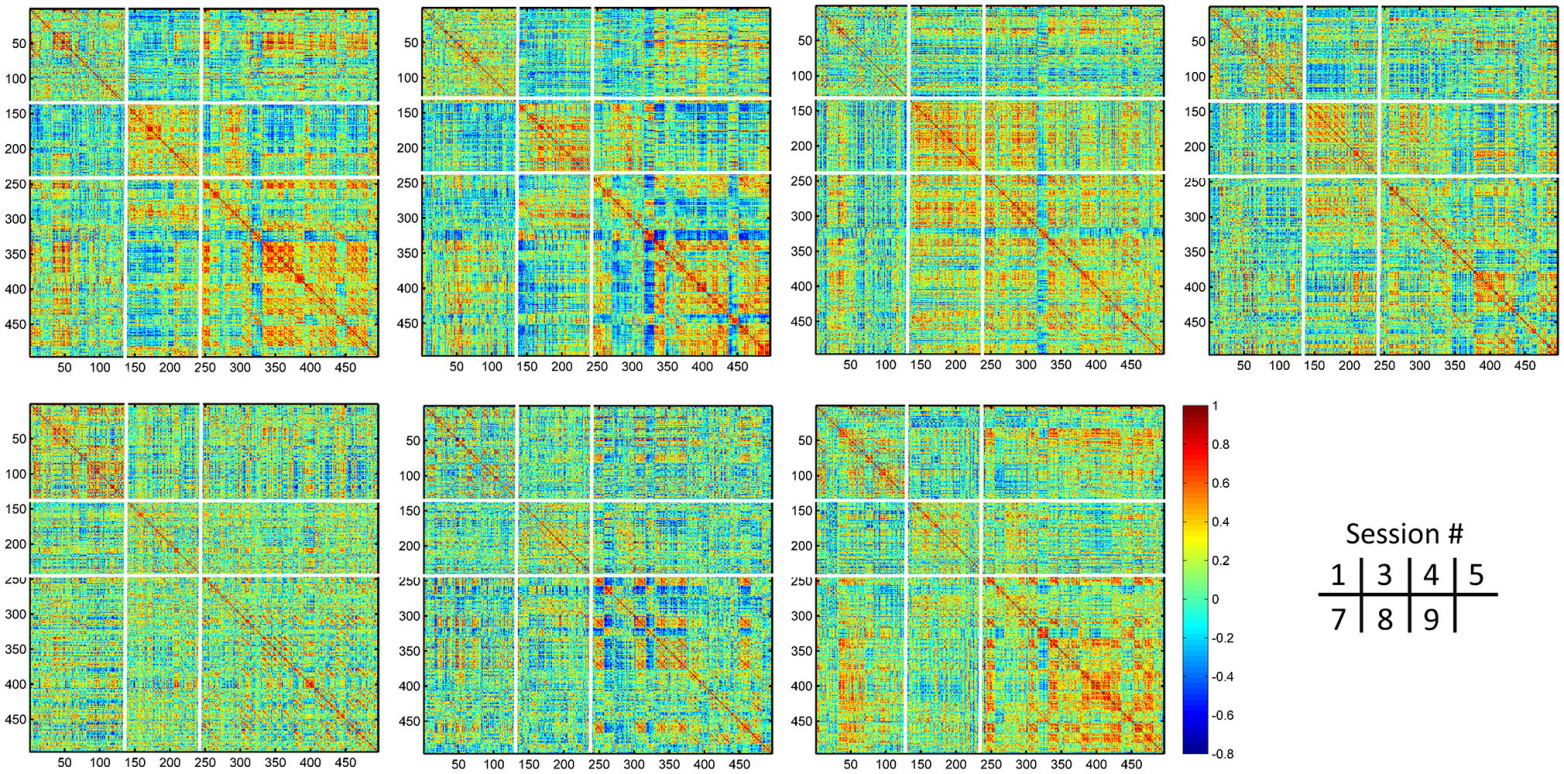
Individual dHOFC matrices of the same subject for all 7 sessions. The dHOFC matrices for the high-level cognition-related networks (fronto-parietal task control and salience networks) from a randomly selected subject (#9) are plotted. Note this is the same subject for demonstration the reliability of dHOFC within the primary functional system in Figure 9. The parcellation of the dHOFC matrix is based on the different types of the hypernodes (see Figure 10).

Different from the dHOFC in the primary functional system (Figure 7), the dHOFC of the two high-level functional systems show meaningful and visually detectable and systematic differences in the test-retest reliability, which becomes more prominent when only looking at the connections with fair or better reliability (Figure 10C). That is, for between-network dHOFC, their connectivity strength is weak, and their connectivity reliability is also poor, while the other two types of the dHOFC have both greater strength and better reliability. At the subject level, Figure 12 shows the dHOFC matrices derived from all the seven rs-fMRI sessions of the same subject (subject #9), with a roughly stable pattern.

Compare with the dHOFC in the primary functional system, those in the high-level functional systems have *better* reliability for several (mainly the between-network and the modulatory) connections while lower reliability for several other (mainly within-network) connections (Figure 8C). When only looking at the strong and putative connections, dHOFCs in the high-level system are more reliable (Figure 8D), with more (66.4%) connections characterized as fairly reliable or better.

### Sliding window length significantly affects dHOFC reliability

**Figure 13:**
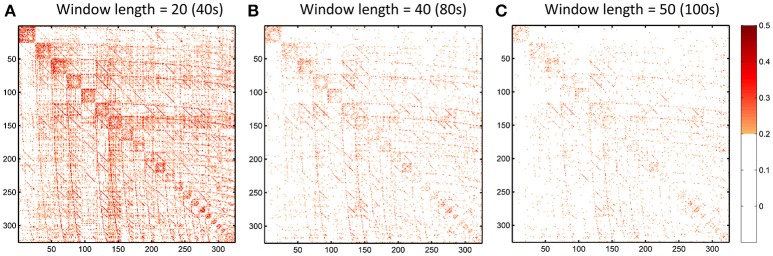
ICC of the dHOFC calculated based on different window length settings (40s, 80s, and 100s in panels **A–C**, respectively). The dHOFCs in the hand sensorimotor areas (the primary functional system) are shown. The dHOFC links with ICC > 0.2 (fair reliability or better) are indicated in orange-to-red colors.

**Figure 14:**
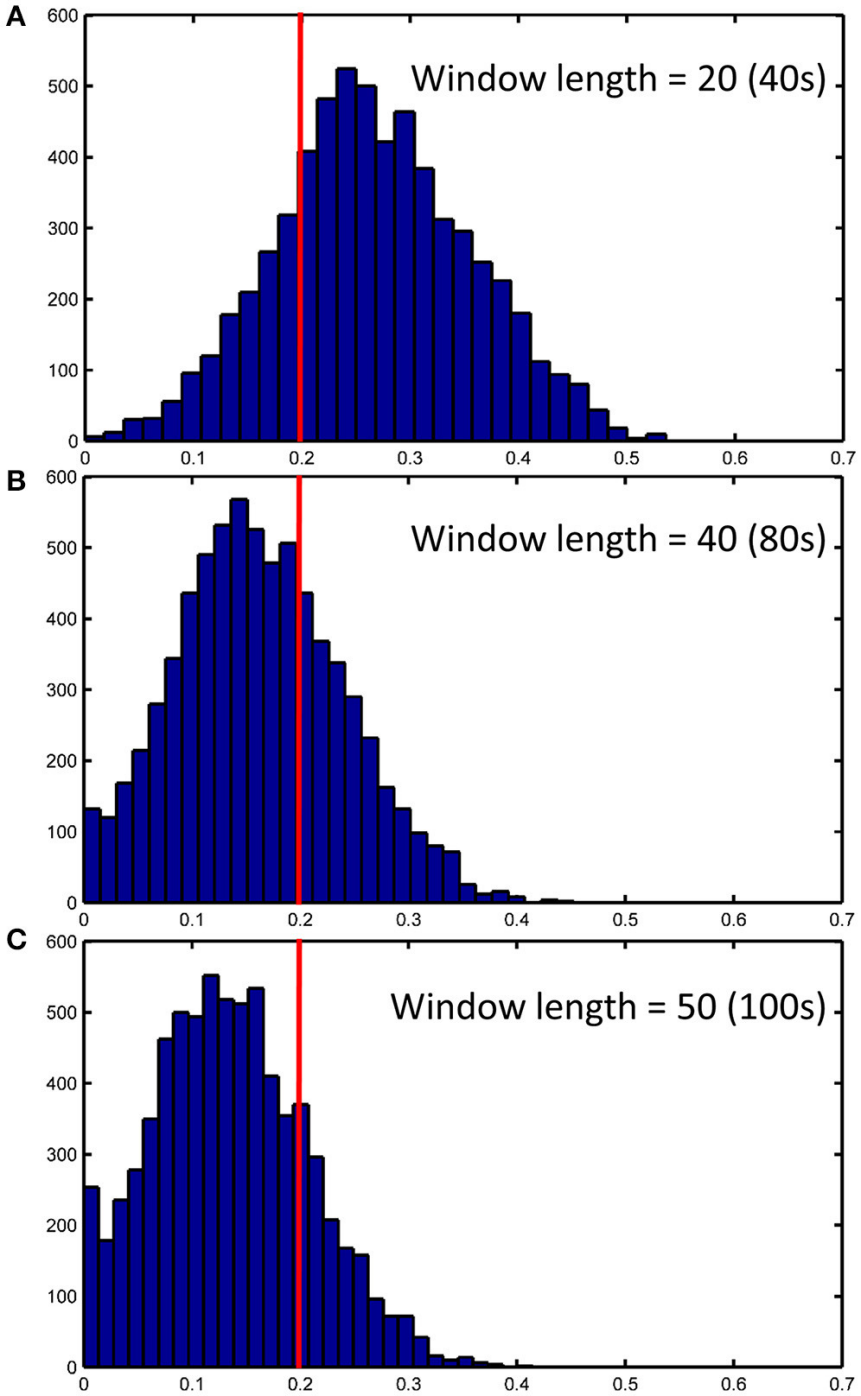
ICC distributions for all strong dHOFC links with different window length settings (40s, 80s and 100s in panels **A–C**, respectively). The dHOFCs in the hand sensorimotor areas (the primary functional system) are used. The strong dHOFC links are defined as those having group-averaged dHOFC > 0.36. The red line indicates an ICC threshold of 0.2 (thus, the right side of this red line indicates fair or better reliability).

We further show how the length of sliding window (or the window width), an important parameter for both dynamic LOFC and dHOFC analyses, will affect dHOFC reliability. The ICC matrices based on different window lengths of 40, 80, and 100 s are shown in Figure 13. Together with the main dHOFC ICC result using a window length of 60 s (Figure 7C), we, for the first time, revealed that the setting of sliding-window length significantly affected the dHOFC test-retest reliability. Shorter window length generated better reliable dHOFC. Based on the ICC values, the window length of 40, 60, 80, and 100 produces 77.3, 49.5, 30.3, and 18.9% fairly reliable (ICC > 0.2) dHOFC links among all the strong dHOFC links (Figure 14).

We still chose the window length setting of 60 s as the main dHOFC result, because the previous comprehensive simulated experiments have shown that a too short window length setting may cause limited sample size in the calculation of dynamic LOFC within each window and could *overestimate* the dynamic FC. In other words, with small window length, we may inflate the window-based LOFC estimations and increase the possibility of type-I error in finding the significant dynamic FC. This will, in turn, compromise the dHOFC calculation because dHOFC is based on the second round of the correlation analysis on the dynamic LOFC time series, and the overestimated LOFC changes may cause bias in the following dHOFC calculation.

## Discussion

### General discussion

In this paper, we assessed the test-retest reliability of all existing HOFC (high-order FC) metrics extracted from young healthy adults. Table 1 summarized all definitions and potential biological meanings for all the HOFC metrics involved. We found that, in general, all the methods have acceptable test-retest reliability. Please also see Table 1 for a summary of all reliability assessment results, and Figure 11 for specially summarized connectivity strength and reliability characteristics for different types of the dHOFC links. The goal of presenting such reliability analysis results is to obtain new knowledge based on the reliability analysis for better understanding the biological meaning of different types of HOFC, deriving guidance for future HOFC studies, and accelerating wider clinical applications using HOFC.

To our best knowledge, there is no such reliability study before on the HOFC metrics. We note that there is a recent study investigating the reproducibility of dynamics LOFC-based brain transient status detection across different data sets (Abrol et al., 2016), which suggested that a few transient LOFC patterns are reproducible; but this study didn't go further to analyze the high-order FC and its reproducibility. Here, we use a dedicated dataset with amply repeated scans and sample size to produce an accurate estimation of the HOFC's test-retest reliability. We believe that this novel test-retest reliability studies on such state-of-the-art connectomic metrics could have instructive meanings toward understanding how the human brain is functionally organized.

### Why focusing on HOFC's test-retest reliability?

Besides characterizing pair-wise temporal synchronization of rs-fMRI BOLD signals and building such traditional LOFC brain networks, researchers are also eager to look for the methods that can capture more complex functional organization of the human brain, i.e., HOFC. The HOFC may have more generalized definition, as long as it captures more complex functional organization, e.g., hierarchical FC architectures (Cordes et al., 2002), modularity/rich-club from deep analysis to the LOFC networks (van den Heuvel and Sporns, 2011), hypergraph consisting of hypernodes and hyperlinks (Jie et al., 2016), cross-modality association (Honey et al., 2009) and context-sensitive divergence (Hermundstad et al., 2013), but here we only focus on the narrowly defined HOFC metrics, which are the metrics that have been explicitly proposed to be “high order” based on “correlation's correlation.” Of note, a previous study first calculated dynamic local LOFC and then calculated regional covariance of the regional dynamic local LOFC time series (Deng et al., 2016), which is somewhat also based on the correlation of correlations. We think that this method is more like the dHOFC, but still characterizing the pairwise relationship since the first round of correlations are collapse into regional time series. Although this paper did not provide reliability or reproducibility results, it did show a highly structured high-level functional organization. Another recent work also calculated topographical LOFC profiles (Zhang J. et al., 2016) and their dynamics, but they further calculated the similarity among each brain region's topographical LOFC profiles across time to define a variation-based metric for each brain region. Therefore, they did not use inter-regional topographical similarity to define HOFC but rather using intra-regional time varying topographical information to capture brain function. All these state-of-the-art studies have indicated that characterizing high-order brain functional organization is the common research interest and also a hot topic. Therefore, test-retest reliability on these HOFC metrics is highly necessary.

Of all the studies which explicitly defined or adopted HOFC, the *tHOFC* characterizes similarity of the topographical LOFC profile between any two brain regions; the *aHOFC* defines a different pair-wise topographic profile similarity which is actually a cross-level (i.e., the modulation between the low-level and the high-level FC organizations among brain regions) HOFC measurement; and the *dHOFC* defines an even more complex, i.e., four region-based functional relationships by adopting dynamic LOFC profiles, where the covariance of two LOFC dynamic time series naturally reflect a modulatory interaction. Based on the network belongingness of every four brain regions, we have the opportunity to *explicitly* define different types of high-level modulation rather than just *inherently* considering such high-level functional coherence like most of the existing LOFC dynamics studies on brain “status.” In summary, all the HOFC metrics are methodologically innovative and state-of-the-art. Most importantly, these metrics may characterize different aspects of biologically meaningful functional organization architecture, which is systematically different from LOFC. In order to further validate this argument, we need to assess their reliability to add further support to this hypothesis.

### Reasons and factors that may cause variation in HOFC reliability

There are several factors that could cause the difference in reliability among the HOFC metrics. Next, we will discuss the possible contributing factors that may lead to such differences in HOFC reliability, which include:

- *Various types of noise and artifacts* (e.g., cardiac pulsation and head motion) in the rs-fMRI data may interfere LOFC estimation (Chang and Glover, 2009; Power et al., 2014), which often leads to overestimated LOFC due to the structured and spatially overspread noise. Since HOFC is calculated based on LOFC, the noise and artifacts can interfere HOFC as well, although the effect will be different for HOFC compared to LOFC.
- *The complexity of the algorithm*. First, noise problem can be exaggerated when there are more operations (“correlation's correlation”) applied on the data. In other words, the noise-induced error may propagate and increase by further steps of correlation analysis. Similar substantial reliability reduction has been witnessed in the previous reliability studies on the graph-theoretic analysis-derived network properties from the LOFC network (Wang et al., 2011). Moreover, dHOFC has several freely estimable parameters, one of which is the sliding window length. From the Figure 7C and Figures 13–14, we can see that window length indeed affects dHOFC reliability (with the shorter window length leading to more reliable dHOFC). Therefore, if using dHOFC to detect potential disease biomarkers, we may *not only* have a risk in the reliability reduction due to the computational complexity, *but also* have to decide the optimal parameter setting. On the other hand, tHOFC and aHOFC do not have free parameters as long as the region-averaged rs-fMRI signals are obtained.
- *The HOFC strength itself*. An interesting finding for all types of HOFC (and the LOFC previously) is that, generally, connections with greater strength may be more reliable, and vice versa. Such a phenomenon is more prominent for the dHOFC. This may be because weaker connectivities are more likely to be affected by the noise and artifacts. Of note, it is difficult to determine the threshold for weak/strong dHOFC as the parametric testing, such as the *t*-tests tends to overestimate the “significant” dHOFC, i.e., even a small dHOFC could be significantly large due to a large number of sliding windows and the statistical dependence among nearby windows. Based on the suggestion of previous dynamic FC study (Leonardi and Van De Ville, 2015), even a large dynamic FC could be purely induced by noise. Thus, we use a relatively large threshold to determine strong dHOFC (>0.36). In future, non-parametric analysis, such as permutation test can also be used to generate the “null model” of dHOFC and determine which is significantly strong. Here, to make fair comparison among different window lengths, we use the predefined threshold of dHOFC > 0.36 to identify strong dHOFC links. However, such a rule does not apply to several LOFC, tHOFC and aHOFC links, such as the connections among the DMN, FPN, and SN; interestingly, their weak connectivities are astonishingly stable across repeated scans (see Figures 4–6).
- *The subject's varying status*. Recently, studies on brain LOFC dynamics have revealed that the brain functional network is not a static but a continuously changing system (Hutchison et al., 2013; Calhoun et al., 2014; Preti et al., in press). Decompositions to the LOFC spatiotemporal dynamics have revealed a few instantaneous LOFC network patterns that occur from time to time and switch to each other with certain transformation probability, which may represent different brain “statuses” (Allen et al., 2014). The occurrence frequency and the dwelling time of the status may be substantially different in different rs-fMRI sessions; moreover, several statuses may not occur at all during a particular scanning session (Abrol et al., 2016). Such a variation could be larger if the interval of the repeated scans is longer. Although our test-retest data were acquired within a month, such a period will still allow unneglectable changes in subject's physical and mental conditions (e.g., drowsiness) to happen and lead to differences in status switching and their occurrence frequency. Since HOFC is proposed to measure high-level brain functional architecture, a small variation may still affect its reliability. We think that dHOFC could be affected more because this metric *per se* is directly estimated on the basis of dynamic analysis.
- *Head motion*. Although we had stringently controlled head motion effect according to the strict data inclusion criteria, head motion can still be a source of the reduced reliability. We believe that the head motion will have more effect on dHOFC estimation because sliding window-based analysis uses fewer samples to conduct temporal correlation, such that the robustness to the head motion-related artifacts could drop. This argument has been supported by both previous studies (Laumann et al., 2016) and the leftward shift of the ICC histogram from Figure 4 to Figures 8A,C.
- *Other unavoidable factors*, such as the changing condition and status of the MRI scanner, will likely to affect the test-retest reliability.

### Biological meaning of HOFC indicated by reliability assessment result

Based on reliability analysis, we may have a chance to revisit the underlying biological meaning of the HOFC. Our result has four major implications. First, we examined which HOFC links have reliability gain when comparing tHOFC (and aHOFC, with the similar result) and LOFC. We found that the links with better reliability than those of the LOFC are highly *structured* with highly *specified* anatomical location. Most of them are the inter-network connectivities between the high-level and the primary functional systems (Figure 5C). The primary systems are the sensorimotor and visual areas, while the high-level functional systems include the DMN, FPN and SN, which have a perfect agreement with so-called “triple networks” (Menon, 2011). The triple networks have been proposed to be responsible for high-order cognitive functions, such as task control, attention, self-awareness, etc. Meanwhile, many neurological and psychiatric diseases (such as AD and schizophrenia) have abnormalities commonly located at such three networks. The increment of test-retest reliability for the tHOFC and aHOFC indicates that the *t*HOFC can *more reliably* estimate the connections between the high-level and low-level brain networks. These results support the previous finding using the tHOFC, that is, the topographical LOFC profile can suppress noise in several links (Zhang H. et al., 2016). Because these “reliability enhanced” links are mostly the weak connections, if the noise level is not favorable, these connectivities cannot be used for biomarker detection and disease classification due to the noise-induced reliability reduction. Our result suggests that tHOFC and aHOFC could be more suitable for such studies if these particular (although weak) connections are of interest. From another viewpoint, this result indicates that tHOFC and aHOFC are able to model the feedforward and feedback functional relationships, which may reflect information exchange between the high-level and the primary areas.

Second, after visualizing the extent of the reliability gain for each brain region, we found that the mostly benefiting nodes are the medial frontal regions in the DMN and the lateral frontal regions in the FPN and SN (Figure 6B), indicating the importance of these areas in such a cross-level information exchange. Moreover, we, for the first time, show that these medial and lateral frontal regions could be functionally important based on the reliability gain against LOFC. In the future, more efforts should be made on these putative but weak high-order cross-level interactions between the triple networks to the primary functional areas. The importance of such a type of HOFC links could be diminished if only traditional LOFC is used. Based on this finding, we have a further tentative assumption that, for the neurodegenerative diseases, such as AD and the neurodevelopmental disorders, such as autism spectrum disorder, at the very beginning, the pathological attack (such as neurofibrillary tangles and amyloid beta-peptide deposition in AD) could first occur at these frontal areas (Braak and Braak, 1991). At this early stage, there is usually no significant cognitive abnormalities for the patients. *We hypothesize that it is such high-order cross-level feedback and feedforward connections that could be affected at this period, and the high-level to primary information exchanges are likely to be already changed*. Traditional LOFC is less reliable for such connections, thus early detection is difficult and less sensitive. If tHOFC, especially aHOFC, is used as connectivity-based metrics, we could have much larger chances to detect such early but subtle changes.

Third, as shown in Figure 7A, the group-level dHOFC matrix in the hand sensorimotor system shows the prominent modular structure (i.e., small blocks along the main diagonal of the dHOFC matrix). The dHOFC strength within modules is higher than that between modules. Further investigation revealed that the higher dHOFC in each of the block or module had a brain region acting as a common driving source, so that any dLOFC links sharing the same driving region had quite similar dynamic patterns along time. For example, the dHOFC among dLOFC_12_, dLOFC_13_, …, dLOFC_1R_ (which all share the region #1) are stronger than the dHOFC between dLOFC_12_ and dLOFC_34_ (because they share no region). This could indicate the organization architecture of the dHOFC in the sensorimotor system; that is, many strong dHOFC hypernodes (dLOFC links) share a common driving source from a single brain region and this can form a “star-shaped” local topological structure. This star-shaped cluster could be the basic unit for high-level brain functional organization. Traditionally, it is impossible to reveal such a high-level spatiotemporal organization architecture.

Fourth, in this study, we have included two high-level functional systems (FPN and SN) for dHOFC analysis. The reliability matrix has shown a structured and inherently well-organized pattern (Figures 10B,C), consistent with the pattern of the dHOFC strength (Figure 10A). Based on the complexity of the dHOFC's definition (involving four regions for characterizing a hyperlink of dHOFC), we have further separated dHOFC hyperlinks into the *within-network, between-network* and, completely new, *modulatory* types (containing hybrid inter-network connection(s) as hypernode(s); see Figure 11C). Note that, previously, there is no study on the third type of the connections. We found that the between-network dHOFC, which consists of two intra-network hypernodes for each of the two networks, respectively, are nearly zero (weak connections). This result indicates that the two high-level functional systems, as shown by their respective nearly *uncorrelated* dynamic connectivity profiles, may work quite *independently*. The reliability of such type of dHOFC is also poor, meaning that such weak high-order connectivities are prone to be affected by noise. However, the within-network dHOFC, similar to previous findings for the within-network LOFC, is relatively strong and much more reliable than the between-network dHOFC or LOFC. The most interesting finding is that the *modulatory* dHOFC, especially when both hypernodes are inter-network connections (with the two ROIs of each hypernode belonging to two different functional systems), are also relatively strong with better reliability. This result indicates that the brain functional organization is not in a one-by-one or pairwise manner. The two high-level functional networks may *not only* interact with each other via pairwise LOFCs, *but also* have extensive and deep modulatory relationship in a high-order way. Such a high-order relationship can be further divided into two subtypes (Figure 11C), reflecting different modulatory interactions. In this sense, the dHOFC may be able to model more complex interactions among the brain networks that cannot be easily modeled using the traditional inter-network LOFC.

**Figure 15:**
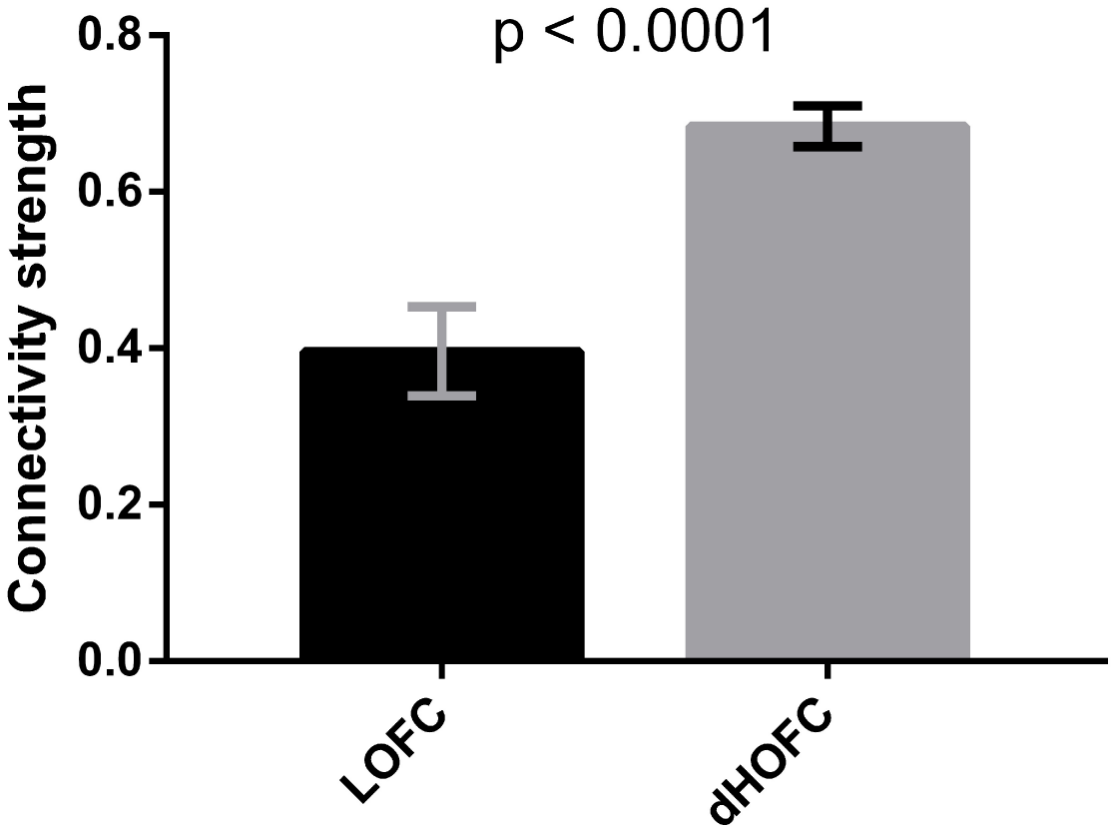
Comparison between inter-network LOFC and modulatory dHOFC. The inter-network LOFC shown in the first bar are the largest 50 LOFC links between fronto-parietal task control and salience network. The modulatory dHOFC shown in the second bar are the largest 50 dHOFC links for the third category of three different types of dHOFC (i.e., with both hyper-nodes being the inter-network connections). Error bar shows the standard deviation. The *p*-value is derived from non-parametric group difference test (Mann-Whitney test, two-tailed).

Finally, as shown by Figure 10A, there are strong off-diagonal connections for the case 1 of the modulatory dHOFC, indicating that the two high-level cognitive function-related networks indeed communicate with each other more in a more complex manner than any LOFC can capture. However, when compared the connectivity strength of the similar off-diagonal LOFC (i.e., the mean inter-network LOFC between the FPN and SN), for the strongest 50 connections, we found that the dHOFC values are significantly (*p* < 0.0001) larger than LOFC (Figure 15). Moreover, such a type of the dHOFCs has acceptable reliability. Therefore, we propose that the modulatory dHOFC with each of the two hypernodes connecting with both networks (see Figure 11C, case 1) can better characterize inter-network functional association via complex high-order modulatory interactions. In the future, this type of dHOFC could be specifically selected as features to search for potential biomarkers of brain disease if inter-network connectivity is the main target.

### Suggestions and guideline to future HOFC study

Based on the findings on HOFC reliability, we give several suggestions to future studies that focus on high-order brain functional organization or its modulation by different experimental states or diseases:

- The tHOFC and aHOFC may have less reliability for within-network connections than LOFC, but are still moderately reliable. If interested in the within-network connectivity, it's better to use LOFC.
- The tHOFC and aHOFC have higher reliability for the weak between-network connections. If interested in such type of connections, it's better to use tHOFC or aHOFC. The tHOFC seems to have *both* acceptable reliability for within-network connectivity *and* more reliable between-network connectivity. Due to such a trade-off, the tHOFC may be more suitable for the exploratory whole-brain network analysis, including both within- and between-network connectivities.
- The aHOFC is especially reliable for modeling high-level feedback and feedforward relationship between the high-level cognition-related and the primary functional systems, which is suitable for studies on top-down or bottom-up connectivities.
- dHOFC implementation should be careful due to its lower reliability compared with that for static LOFC or HOFC. However, within-network and modulatory dHOFC or relatively strong dHOFC are still sufficiently reliable. Future dHOFC studies should focus on these dHOFC links.
- Data processing parameters, such as sliding-window length should be carefully determined for dHOFC calculation. Too small window length may be less robust to noise and may lead to spuriously high “reliability.” A window length of 60 s is a recommended choice for robust dHOFC estimation with adequate reliability.
- For early diagnosis studies, in order to increase detection sensitivity, it's better to choose a certain type of HOFC to characterize the subtle connectivity abnormalities. For example, weak connections might be more likely to be affected by the pathological attacks than strong connections; all the HOFC metrics have satisfactory reliability for the weak connections.
- While static LOFC does not have adequate sensitivity for biomarker detection, modulatory dHOFC, especially the case 1, could be an alternative approach to estimate those deeply inherent inter-network interactions.

## Limitations and future works

First, in this paper, we only focused on the reliability assessment of the connectivity strength without going further to assess the reliability of graph-theoretical analysis-based network properties, which we think deserves a dedicated research after more suitable complex network construction approach for the HOFC is proposed. Second, this paper is dedicated to investigating HOFC reliability, the further study on the biological relationship (validity) between the HOFC strength and neurocognitive measurements or disease states are not our main goal and will be investigated in the future. Third, the test-retest reliability with varied inter-scan interval (especially the intra-session reliability) will better disentangle the mixed effect of influencing factors on the HOFC reliability. This is especially important for the dHOFC, because it is based on the dynamic LOFC which is theoretically expected to be fluctuating. Although the dHOFC calculates the coordination of the dynamic LOFC, making this HOFC metric more like a measurement of “trait” than “state,” a dedicated study on how the changing brain “state” may affect the trait characterization is highly required. Finally, due to the increased dimensionality, we only calculate dHOFC for a few functional systems. In the future, the better algorithm needs to be proposed to overcome such a limitation and extend our understanding of the neurobiological meaning of the dHOFC in the whole-brain level.

## Author contributions

HZ, YZ, and XC analyzed the data. HZ drafted the manuscript. DS conceived this work, led the project, and revised the paper.

### Conflict of interest statement

The authors declare that the research was conducted in the absence of any commercial or financial relationships that could be construed as a potential conflict of interest.
